# Redox- and Photo-Responsive Fe^3+/2+^-Cross-Linked Carboxymethyl Cellulose Methacrylate Dissipative Gels: Synthesis and Applications

**DOI:** 10.1021/acsami.6c07915

**Published:** 2026-06-16

**Authors:** Jianghe Zhao, Yunlong Qin, Muhammad Abdel-Haq, Vitaly Gutkin, Ehud Neumann, Abraham J. Domb, Rachel Nechushtai, Yuwei Hu, Junji Zhang, Gilad Davidson-Rozenfeld, Itamar Willner

**Affiliations:** † Institute of Chemistry, The Center for Nanoscience and Nanotechnology, 108405 The Hebrew University of Jerusalem, Jerusalem 91904, Israel; ‡ Faculty of Medicine, School of Pharmacy, The Hebrew University of Jerusalem, Jerusalem 9112002, Israel; § The Harvey M. Krueger Family Center for Nanoscience and Nanotechnology Edmond J. Safra Campus, The Hebrew University of Jerusalem, Jerusalem 9190401, Israel; ∥ The Alexander Silberman Institute of Life Sciences, The Hebrew University of Jerusalem, Jerusalem 9190401, Israel; ⊥ School of Chemical Engineering and Technology, Sun Yat-Sen University, Zhuhai, Guangdong 519082, P. R. China; # Key Laboratory for Advanced Materials and Joint International Research Laboratory of Precision Chemistry and Molecular Engineering, Feringa Nobel Prize Scientist Joint Research Center, Frontiers Science Center for Materiobiology and Dynamic Chemistry, Institute of Fine Chemicals, School of Chemistry and Molecular Engineering, 47860 East China University of Science and Technology, Shanghai 200237, China; ∇ Food Science Department, Tel-Hai Academic College, D.N. Upper Galilee, Kiryat Shmona 12210, Israel

**Keywords:** Controlled drug-release, Self-healing, Soft-robotics, Transient gel, Smart gel, Cryogel

## Abstract

The synthesis of redox-active Fe^3+^-cross-linked carboxymethyl cellulose methacrylate cryogels (**Fe** ^**3+**^ **-CMCMA**), revealing switchable, transient, dissipative stiffness properties, is introduced. The switchable, transient functions are triggered by ascorbate-mediated reduction of the high-stiffness gel to the lower-stiffness Fe^2+^-CMCMA gel, and the concomitant aerobic oxidation of the Fe^2+^-CMCMA to the Fe^3+^-CMCMA gel state. Alternatively, integration of native photosynthetic photosystem-I (**PS-I**) into the cryogel matrix allows the photosensitized reduction of the Fe^3+^-CMCMA cryogel to the lower stiffness state Fe^2+^-CMCMA, and the concomitant, temporal, aerobic recovery of Fe^2+^-CMCMA to the high-stiffness Fe^3+^-CMCMA gel. The transient dissipative stiffness of the Fe^3+/2+^-CMCMA cryogel is characterized by temporal rheometry, dynamic stretching stress–strain experiments, XPS, and SEM imaging. The transient chemical/light-triggered stiffness functions of the Fe^3+/2+^-CMCMA, or PS-I-integrated Fe^3+/2+^-CMCMA gels are applied to develop transient self-healing matrices, and frameworks for cyclic, transient release of loads integrated in the gel matrices. This is exemplified by the ascorbate/light-induced cyclic transient release of tetramethyl rhodamine-dextran (**TMR-D**), insulin, and the anti-VEGF aptamer loads. In addition, the stimuli-responsive Fe^3+/2+^-CMCMA cryogel is used to construct a bilayer-soft-robotic bending device. Bilayer composite consisting of thermoresponsive poly-*N*-isopropylacrylamide (**pNIPAM**)/Fe^3+/2+^-CMCMA, or pNIPAM/PS-I-loaded-Fe^3+/2+^-CMCMA devices are assembled. Switchable, reversible, and transient, thermal and chemical/light-triggered bending of the bilayer devices are demonstrated.

## Graphical abstract

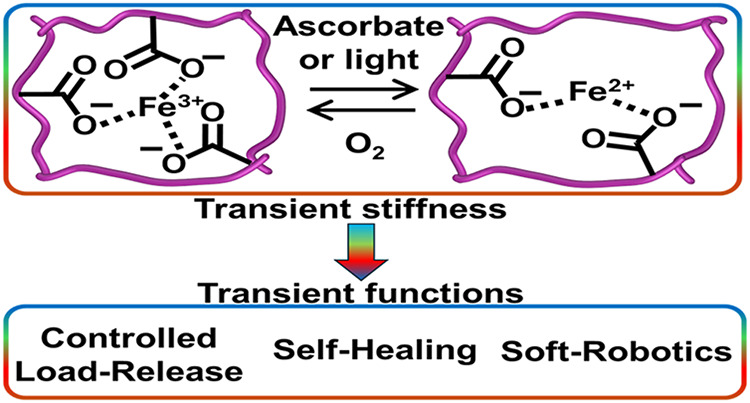

## Introduction

The synthesis of stimuli-responsive hydrogels attracts substantial research efforts.^1–5^ Diverse physical and chemical triggers were applied to reversibly switch hydrogels properties. Physical triggers included temperature,^6,7^ light,^8–10^ electrical,^11–13^ magnetic field,^14,15^ or ultrasound.^16,17^ Chemical triggers included pH,^18–20^ chemical agents,^21–23^ metal ions,^24–26^ redox agents,^27–29^ or supramolecular complexes stabilized by H-bonds,^30,31^ donor–acceptor interactions,^32,33^ or host–guest complexes.^34–36^ The control over the stiffness and elasticity properties of the stimuli-responsive gels attracted special interest,^37–39^ as it enabled triggered, switchable reconfiguration of the gels between defined stiffness states,^40–43^ facilitating switchable mass transport of substrates between the gel frameworks and the bulk solution.^44,45^ Besides controlling the gel’s properties through the chemical composition of the matrices, the methods used to synthesize the gels also dictate their properties. Specifically, gels prepared at freezing conditions, cryogels, attract growing interest.^46–49^ The cryogel, prepared by analog procedures to hydrogels, yet at freezing conditions, leads to crystalline solvent domains coated by concentrated cross-linked polymer matrices that, upon thawing, yield permanent matrices consisting of interconnected solute-loaded macropores in the gel frameworks.^50^ The interconnected macropores introduce distinct different properties into the cryogels as compared to analog hydrogels. These are reflected by differences in mechanical properties, such as the compressibility of the cryogels.^51,52^ In addition, the large interconnected macropores allow convectional transport of the solute (and solubilized substrates) as compared to diffusionally hindered transport in small-pore-sized analog hydrogels.^53^ Recent studies reported on the synthesis of stimuli-responsive cryogel matrices.^44,54,55^ Indeed, enhanced response times of the cryogels, as compared to analog hydrogels, toward controlled release, catalytic biotransformation, and soft robotic applications were demonstrated.^19^ Diverse applications of stimuli-responsive hydrogels and cryogels were reported for sensing,^56^ controlled drug-release,^57–59^ self-healing,^60–63^ tissue engineering,^64–67^ shape-memory,^68–72^ and soft robotics.^73,74^ Within the family of stimuli-responsive gels, a subclass of gels undergoing dissipative transient stiffness properties attracts growing recent research interest.^75–77^ Dissipative gel frameworks revealing dissipative stiffness properties undergo a stimuli-triggered transition to a lower or higher metastable stiffness state, yet the framework includes an intrinsic mechanism that degrades the metastable, out-of-equilibrium state, resulting in a temporal (transient) recovery of the original parent gel framework.^78,79^ For example, the synthesis and characterization of a Fe^3+^-cross-linked carboxymethyl cellulose (Fe^3+^-CMC), revealing transient, dissipative stiffness properties, has been recently reported.^80^ In this system, reduction of Fe^3+^-CMC (high-stiffness state) using ascorbate or photosensitized reduction of Fe^3+^-CMC (using diffusional Ru­(II)-tris-bipyridine as photosensitizer) yielded the lower-stiffness Fe^2+^-CMC gel. Aerobic oxidation of the Fe^2+^-CMC to the Fe^3+^-CMC gel provides, however, the mechanism to recover the higher-stiffness gel, thus establishing a dissipative, transient pathway controlling the stiffness of the gel. By integrating a load into the higher-stiffness Fe^3+^-CMC and triggering the transient ascorbate/light-aerobic mediated stiffness changes of the gel, transient release of the load was demonstrated. In addition, transient robotic bending of a bilayer gel framework was introduced. Nevertheless, the reported dissipative Fe^3+/2+^-CMC was not free of limitations: (i) The gel framework included a single cross-linking element, redox-responsive Fe^3+^/Fe^2+^-carboxylate bridges. Usually, stimuli-responsive gels include two (or more) cooperative cross-linking motives (stimuli/permanent bridges or stimuli/stimuli bridges). The cooperative bridges in gels enable tunable controlled stiffness changes and diverse applications (stimuli-modulated load-release, self-healing, and shape-memory). Indeed, the reported Fe^3+/2+^-CMC gel demonstrated relatively small stiffness changes with limited applicability. (ii) The preparation of the Fe^3+/2+^-CMC gels lacked a permanent covalent cross-linking motif, generated, for example, by a radical-initiated polymerization. Introduction of a cooperative cross-linking element is not only important for enhancing the switchable complexity of gels and their application diversity, but also for developing new methods to enable dissipative gels on surfaces or to synthesize dissipative microgels. (iii) The photoresponsive Fe^3+^-CMC gel used a diffusional photosensitizer (Ru­(II)-tris-bipyridine) to trigger the photosensitized reduction of the higher-stiffness gel. For any practical application, e.g., ocular drug release, integration of biocompatible, visible-light-triggered photosensitizers, in a nonleakable configuration, in the gel matrix, is essential.

Here, we report on the synthesis of Fe^3+/2+^-cross-linked carboxymethylcellulose methacrylate cryogel matrices (Fe^3+/2+^-CMCMA), undergoing redox-triggered, switchable, and transient stiffness changes. The chemical (ascorbate) or photosensitized reduction of the Fe^3+^-CMCMA gels to the Fe^2+^-CMCMA state and the concomitant aerobic oxidation of the Fe^2+^-CMCMA gels yield stiffness-controlled dissipative, transient gel matrices. Photosystem I (PS-I), extracted from the native photosynthetic apparatus, is integrated as a biocompatible, nonleakable photosensitizer into the gel framework that acts as a photosensitizer controlling the transient stiffness properties of the gels. The transient Fe^3+^-CMCMA → Fe^2+^-CMCMA → Fe^3+^-CMCMA transitions are characterized by spectroscopic (XPS), microscopic (SEM), and mechanical methods (rheometry, tensiometry). The application of the dissipative, transient Fe^3+/2+^-CMCMA gel for transient, dose-controlled release of loads, self-healing, and soft-robotic bending is introduced.

## Materials and Methods

All reagents were obtained from commercial sources and used without further purification unless otherwise noted. Further experimental details describing the methods to prepare the different cryogels and hydrogels, loaded and PS-I co-loaded cryogels, and the different bilayer constructs are described in the Supporting Information. Additionally, methods used to characterize rheometric features, spectroscopic analysis, and bending curvature are addressed.

## Results and Discussion

### Synthesis and Characterization of Fe^3+^-CMCMA Matrices

Figure 1(A) depicts the method to synthesize the Fe^3+^-cross-linked gels (cryogels or hydrogels). The methacrylate functionalized carboxymethyl cellulose, CMCMA, was polymerized by light-induced radical polymerization under freezing conditions (for the preparation of cryogel), or at ambient conditions (to prepare hydrogel). Whenever loading of the gels was required, the loads, e.g., dye, photosystem I (PS-I), protein (insulin), or aptamer (anti-VEGF aptamer) were included in the CMCMA mixture prior to polymerization. The resulting gel was reacted with Fe^2+^ and allowed to be aerobically oxidized to Fe^3+^, resulting in a cooperatively cross-linked Fe^3+^-CMCMA gel.

**Figure 1:**
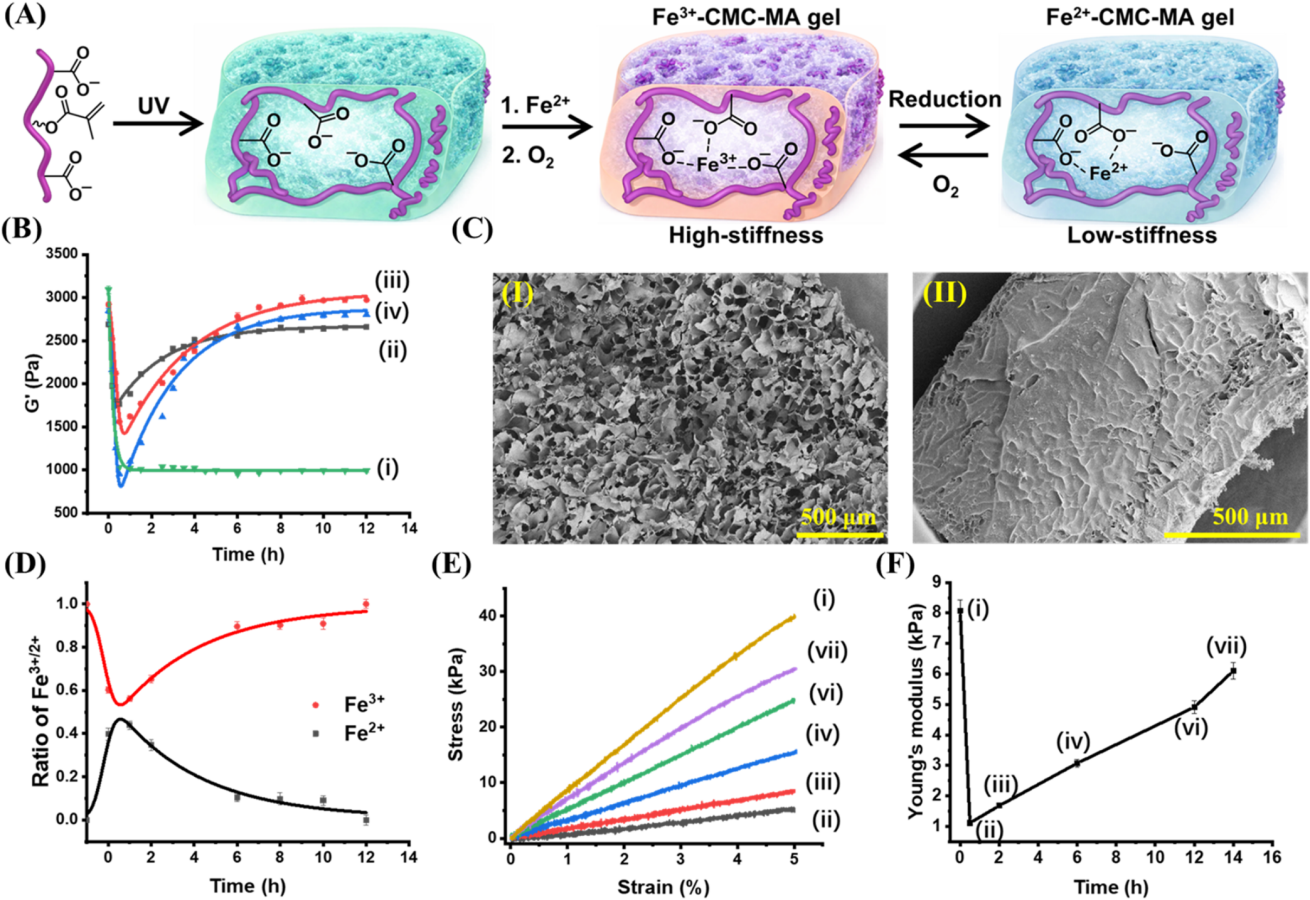
(A) Synthesis of the redox-responsive Fe^3+^-carboxymethyl cellulose methacrylate, Fe^3+^-CMCMA gel, and ascorbate-mediated reduction to the Fe^2+^-CMCMA state that, under aerobic conditions, yield a gel exhibiting transient stiffness behavior. (B) Rheometry, time-dependent G′-changes corresponding to the transient ascorbate-mediated reduction of Fe^3+^-CMCMA to the Fe^2+^-CMCMA gel state, and the aerobic recovery of the Fe^2+^-CMCMA to the parent Fe^3+^-CMCMA state. While curve (i) demonstrates the ascorbate-triggered reduction of Fe^3+^-CMCMA to the Fe^2+^-CMCMA state under N_2_, the reduction under aerobic oxidation in the presence of variable ascorbate concentrations is depicted: (ii) 5 mM, (iii) 10 mM, and (iv) 20 mM. (C) SEM images corresponding to Panel I—Large pore-sized cryogel, and Panel II—Small pore-sized hydrogel. (D) Temporal XPS-evaluated Fe^3+^ and Fe^2+^ ions content upon the transient transition of the Fe^3+^-CMCMA → Fe^2+^-CMCMA → Fe^3+^-CMCMA process. (E) Temporal stress/strain curves corresponding to the transient transition of the Fe^3+^-CMCMA, curve (i), to the Fe^2+^-CMCMA, curve (ii), and back, at time intervals corresponding to (iii) 2 h, (iv) 6 h, (vi) 12 h, and (vii) 14 h. (F) Transient Y-moduli upon the temporal transition of the Fe^3+^-CMCMA state to the Fe^2+^-CMCMA state and back. Y-moduli values correspond to the slope of the curves shown in (E).

Rheometry experiments, Figure S1, demonstrated that the Fe^3+^-CMCMA cryogel *G*′/*G*″ values corresponding to *G*′ = 3092 Pa and *G*″ = 221 Pa, whereas the ascorbate-reduced Fe^2+^-CMCMA cryogel exhibits *G*′ = 1007 Pa and *G*″ = 123 Pa values (while the analog Fe^3+^-CMCMA hydrogel showed *G*′ = 225 Pa and *G*″ = 12 Pa values, and the Fe^2+^-CMCMA hydrogel exhibited *G*′ = 25 Pa and *G*″ = 3 Pa values, Table S1). The higher stiffness features of the Fe^3+^-CMCMA gels, as compared to the Fe^2+^-CMCMA, are consistent with the fact that the Fe^3+^-CMCMA gels are cross-linked by three Fe^3+^-carboxylate bridging units, whereas the Fe^2+^-CMCMA gels are cross-linked only by two Fe^2+^-carboxylate ligand bridges (shown in Figure 1(A)). Figure 1(B) depicts the *G*′ temporal changes associated with the ascorbate-induced reduction of the Fe^3+^-CMCMA cryogel to the Fe^2+^-CMCMA state and the transient aerobic recovery of the Fe^2+^-CMCMA cryogel to the original Fe^3+^-CMCMA state. In these experiments, the Fe^3+^-CMCMA cryogels were reduced by variable concentrations of ascorbate (for a fixed time), yielding the Fe^2+^-CMCMA gel. The ascorbate was washed off, and the temporal aerobic oxidation of the Fe^2+^-CMCMA to the Fe^3+^-CMCMA state was followed, while probing the temporal *G*′ value changes. Upon ascorbate-induced reduction of the Fe^3+^-CMCMA gel, initial *G*′ = 3000 Pa, a relatively rapid decrease in the *G*′ value is observed, and as the concentration of ascorbate increases, the degree of changes in *G*′-value is higher (for example, at an ascorbate concentration of 20 mM, the *G*′ values drop from 2844 to 955 Pa). After washing off the ascorbate with MES buffer under anaerobic conditions and subsequent exposure of the sample to aerobic conditions, a slow recovery of the *G*′ value of the reduced Fe^2+^-CMCMA to its original *G*′ value of the Fe^3+^-CMCMA is observed. The higher the ascorbate concentration reducing the parent Fe^3+^-CMCMA, the lower is the resulting *G*′ value of Fe^2+^-CMCMA, and the longer is the recovery time interval to the parent *G*′ value of the Fe^3+^-CMCMA under aerobic conditions. It should be noted that under anaerobic conditions (under N_2_), the *G*′ value of the reduced Fe^2+^-CMCMA stays unchanged, Figure 1(B), curve (i), indicating that the recovery of the Fe^2+^-CMCMA to Fe^3+^-CMCMA, indeed, proceeds only under aerobic conditions. The results are consistent with the primary ascorbate-driven reduction of Fe^3+^-CMCMA to the lower-stiffness Fe^2+^-CMCMA cryogel, which undergoes a dissipative, transient, aerobic oxidation transition to Fe^3+^-CMCMA. For comparison, Figure S2 depicts the *G*′ temporal changes of the analog hydrogel framework, subjected to the ascorbate-induced reduction process. Evidently, the primary Δ*G*′-changes are smaller (at identical ascorbate concentrations), and the recovery time intervals of the parent *G*′-value are substantially longer. These results are attributed to the large pore-sized interconnected channels present in the cryogel matrices, Figure 1(C), Panel I, as compared to the closed, small pore-sized hydrogel framework, Figure 1(C), Panel II, that allows rapid convectional ascorbate solute/O_2_ solubility/transfer across the cryogel, allowing enhanced and superior transient response times of the cryogel. Accordingly, throughout the paper, unless otherwise stated, the functions and properties of the Fe^3+/2+^-CMCMA cryogel matrices will be characterized. The transient transitions between the Fe^3+^ and Fe^2+^ valencies and their relation to the transient stiffness properties were addressed until now by the temporal stiffness properties of the cryogel in the presence of reducing agents (ascorbate) or oxidation (O_2_). Figure 1(D) depicts the X-ray photoelectron spectroscopy (XPS) studies characterizing the Fe^3+^/Fe^2+^ percentage ratio in the cryogel along the temporal ascorbate/aerobic redox-driven process (for the XPS-quantified content of Fe^3+^ and Fe^2+^ along the transient temporal transitions of Fe^3+^-CMCMA → Fe^2+^-CMCMA → Fe^3+^-CMCMA, see Figure S3 and accompanying discussion explaining the binding energy of Fe^3+^/Fe^2+^ and their quantitative deconvolution). While in the initial cryogel, the Fe^3+^-ion is the major cross-linker, reduction with ascorbate yields a cryogel enriched with Fe^2+^-ions. Subsequently, the ascorbate-washed-off gel led, under aerobic conditions, to a temporal increase in the Fe^3+^/Fe^2+^ ratio that yielded, after a ca. 8 h, the initial Fe^3+^/Fe^2+^ ratio. The time scale of the transient Fe^3+^/Fe^2+^ changes overlays the transient G′-value changes, relating the direct link between the Fe^3+^/Fe^2+^ and the stiffness properties of the cryogel. Figure 1(E) depicts the temporal stress/strain curves corresponding to the Fe^3+/2+^-CMCMA cryogel along the transient redox-induced dissipative process. In these experiments, a cryogel “dog-bone” shaped device was designed. The Fe^3+^-CMCMA cryogel device was stretched to a predefined distance with a constant speed before ascorbate-induced reduction, after reduction with ascorbate, and along the transient aerobic oxidation of the Fe^2+^-CMCMA to the Fe^3+^-CMCMA transition, while the respective stretching distance was tuned to a region that adapts a linear stress/strain relationship. While the force required to stretch the Fe^3+^-CMCMA cryogel is high, the ascorbate-induced Fe^2+^-CMCMA cryogel revealed a substantially lower stretching force, and the transient aerobic oxidation of the reduced cryogel demonstrated a temporal increase in the stretching force, consistent with the formation of the higher-stiffness Fe^3+^-CMCMA cryogel. Since the slopes of the stress/strain curves correspond to the Young’s modulus of the respective gels, Figure 1(F) depicts the temporal Young’s modulus values upon the temporal transient transitions of the Fe^3+^-CMCMA → Fe^2+^-CMCMA → Fe^3+^-CMCMA. That is, the rheometry, XPS, and the tensiometry characterizations of the Fe^3+/2+^-CMCMA correlate well with the macroscopic transient stiffness changes of the bulk cryogel. The transient ascorbate/aerobic-stimulated mechanical stiffness properties were followed by probing the rheometry *G*′/*G*″ over three repeated cycles. The *G*′/*G*″ were unchanged ±3% (Figure S3, Supporting Information), demonstrating mechanical stability for at least three cycles.

### Self-Healing of Ascorbate-Triggered Fe^3+/2+^-CMCMA Cryogel Framework

Figure 2(A), Panel I depicts the application of the Fe^3+/2+^-CMCMA as a self-healing material. The Fe^3+^-CMCMA square-shaped (a) cryogel was reduced with ascorbate to the soft Fe^2+^-CMCMA state (b), the ascorbate was washed off, and the square-shaped cryogel was cut into two pieces (c). The two pieces, physically connected (d), were allowed to recover the Fe^3+^-CMCMA state under aerobic conditions (e). After a time interval of 16 h, the two pieces healed into an intact framework, Figure 2(A), images (f),(g). Physically interlinked Fe^3+^-CMCMA pieces under an inert N_2_ atmosphere did not heal, Figure 2(A), Panel II, images (h)–(m), indicating that the healing process was driven by the transition of the interpiece boundary to the high-stiffness Fe^3+^-CMCMA state. Indeed, stretching force experiments, Figure 2(B), enabled the quantitative analysis of the self-healing process, while identifying limitations associated with the process. In this experiment, the Fe^3+^-CMCMA cryogel was synthesized in a “dog-bone” shape, shown in Figure 2(B), Panel I (a). The device was then reduced to the lower stiffness state, Fe^2+^-CMCMA (b), and cut into two pieces (c). The two pieces were physically connected (d) and allowed to heal under aerobic conditions, yielding the healed Fe^3+^-CMCMA framework (e). Figure 2(B), Panel II, depicts the stretching-induced stress/strain curves of the parent Fe^3+^-CMCMA cryogel before reduction, curve (i), after reduction with ascorbate prior to cutting, curve (ii), and the healed gel generated after 12 h of aerobic transition of the physically interconnected pieces into the Fe^3+^-CMCMA state, curve (iii). The Young’s modulus of the parent Fe^3+^-CMCMA gel corresponds to 7.9 kPa. Reduction of Fe^3+^-CMCMA to the Fe^2+^-CMCMA yields, as expected, a gel of lower Young’s modulus, corresponding to 2.4 kPa. After a time interval of aerobic, redox-induced healing, the healed gel exhibits a Young’s modulus of 6.9 kPa, close to the parent Fe^3+^-CMCMA gel. Nevertheless, the stretching distance of the parent gel state shows a linear stable stress–strain curve up to a distance of 1 mm (5% strain). In contrast, the healed gel revealed a stretching stability up to 0.74 mm (3.7% strain), and at this distance, the healed gel was separated at the healed boundary. Presumably, the boundary-induced defect reflects the limitation of the healing process.

**Figure 2:**
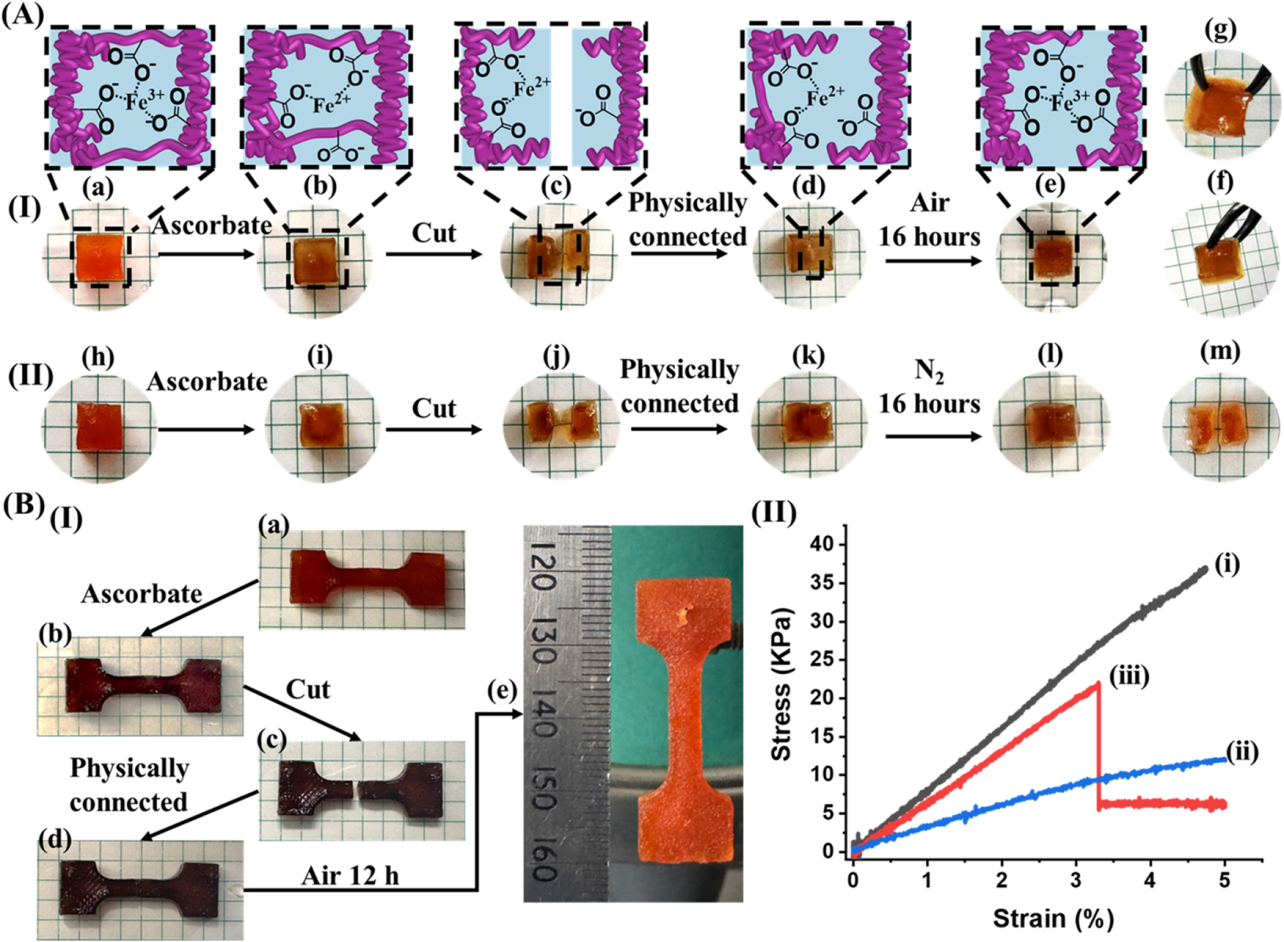
(A) Schematic self-healing of the Fe^3+/2+^-CMCMA gel, and corresponding visual images. Panel I, images (a)–(g)–under aerobic conditions leading to self-healing. Panel II, images (h)–(m)–under N_2_ conditions, prohibiting the self-healing process. (B) Panel I—self-healing experiment of a “dog-bone” gel structure image (a)–(e). Panel II—quantitative evaluation of the self-healing process by monitoring the strain/stress stretching curve of the Fe^3+^-CMCMA before reduction (i), after ascorbate-induced reduction (ii), and after cutting the Fe^2+^-CMCMA device and physical connection of the two parts, allowing their healing under aerobic conditions for 12 h (iii).

### Ascorbate-Induced Transient Load-Release from Fe^3+/2+^-CMCMA Cryogel Matrices

The dissipative, transient, stiffness-controlled properties of the Fe^3+/2+^-CMCMA cryogel matrices were then implemented for the cyclic, dose-controlled release of loads (for the appropriate calibration curves, see Figure S4, and the quantification of the released loads, Figures S5, S6 and Table S2) from the cryogel matrices, as depicted schematically in Figure 3(A). The Fe^3+^-CMCMA cryogel was loaded with tetramethyl rhodamine-modified dextran, TMR-D (M.W. = 70 000 g/mol, λ_ex_ = 550 nm, λ_em_ = 585 nm). Figure 3(B) displays the dose-controlled ascorbate-induced time-dependent release of TMR-D from the Fe^3+^-CMCMA cryogel treated with different concentrations of ascorbate for 5 min and subsequent washing off the ascorbate triggering agent. While no release of TMR-D from the cryogel in the absence of ascorbate occurred, curve (i), indicating that the high-stiffness Fe^3+^-CMCMA prohibits the leakage of the load, in the presence of ascorbate, the temporal release of TMR-D proceeds, and as the concentration of ascorbate increases, the release efficacies of TMR-D are enhanced, curves (ii)–(iv). Across all systems, the load-release process becomes slower and saturates after approximately 4 h. The release efficiency of the load, controlled by the concentration of ascorbate, is consistent with the degree of reduction of the high-stiffness Fe^3+^-CMCMA to the lower stiffness Fe^2+^-CMCMA state. As the concentration of the ascorbate increases, the stiffness of the cryogel decreases, allowing enhanced release of the load. Moreover, the temporal decrease in the load-release process and the saturation levels of the release are rationalized in terms of the transient aerobic transition of Fe^2+^-CMCMA to Fe^3+^-CMCMA that induces a temporal stiffness increase of the framework, leading to the saturated release levels upon transient conversion of the Fe^2+^-CMCMA to the “locked” Fe^3+^-CMCMA state. Figure 3(C) depicts the temporal release rates of the load from the Fe^3+/2+^-CMCMA matrices, derived from the time-dependent release profiles shown in, Figure 3(B) (time-dependent derivatives). Transient temporal release rates are demonstrated, consistent with the transient stiffness changes of the cryogel. Figure 3(D) demonstrates the cyclic and transient load-release functions of the cryogel. In these experiments, after completion of the first transient release cycle, ascorbate is added to the system, demonstrating the reactivation of the second load-release cycle.

**Figure 3:**
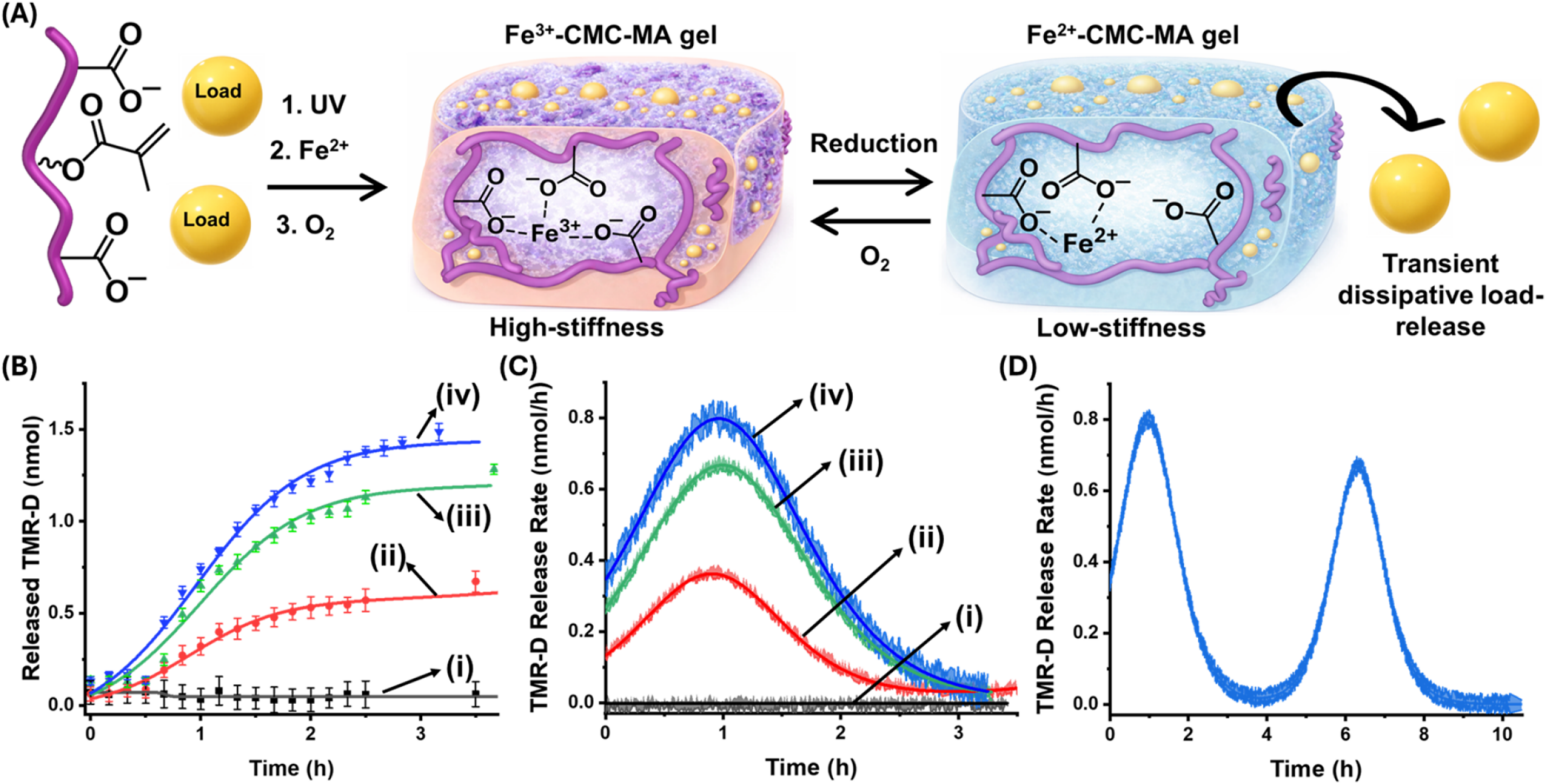
(A) Schematic preparation of loaded Fe^3+^-CMCMA gel and its reduction-induced transient release. (B) Temporal release of TMR-D from the Fe^3+^-CMCMA gel under aerobic conditions in the absence of ascorbate (i), or under aerobic conditions in the presence of different concentrations of ascorbate, corresponding to (ii) 5 mM, (iii) 10 mM, and (iv) 20 mM. (C) Transient release rate of TMR-D from the gel. (i) In the absence of ascorbate (**i**), and under aerobic conditions in the presence of ascorbate: (ii) 5 mM, (iii) 10 mM, and (iv) 20 mM. Transient curves correspond to the time-dependent derivative of the curves depicted in (A). (D) Cyclic transient release of TMR-D from the redox-responsive gel.

The ascorbate-induced dissipative release of loads from the Fe^3+^-CMCMA cryogel was further demonstrated with the transient release of insulin, Figure 4(A), and with the transient release of the anti-VEGF aptamer, Figure 4(B).

**Figure 4:**
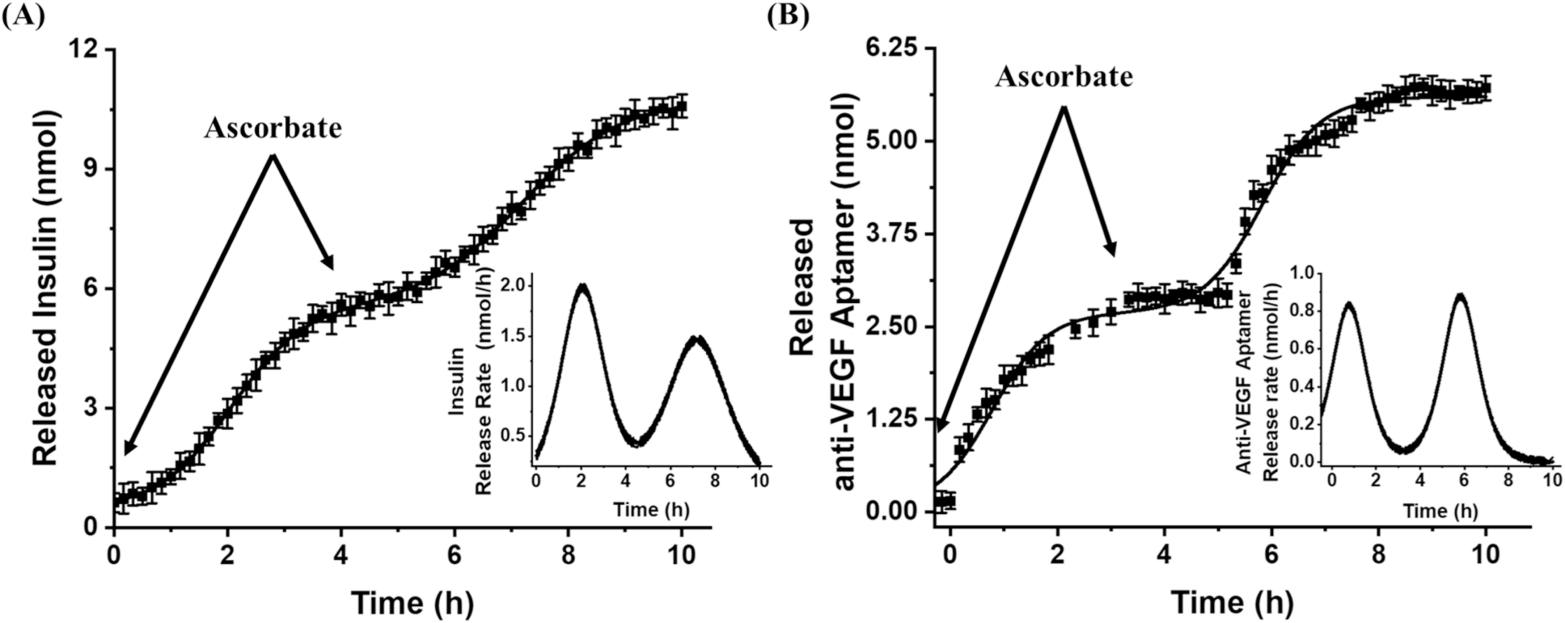
(A) Cyclic temporal ascorbate-mediated release profile of fluorophore-labeled insulin under aerobic conditions upon the triggered redox-mediated transition from Fe^3+^-CMCMA to Fe^2+^-CMCMA, and back to Fe^3+^-CMCMA. **Inset**: Cyclic release rate of the labeled insulin (time-dependent derivative of the release profile). (B) Cyclic temporal ascorbate-mediated release profile of fluorophore-labeled anti-VEGF aptamer under aerobic conditions upon the triggered redox-mediated dissipative stiffness process. Inset: Cyclic release rate of the labeled anti-VEGF aptamer (time-dependent derivative of the release profile).

### Light-Induced Transient Stiffness of PS-I-Loaded Fe^3+/2+^-CMCMA Cryogel Frameworks

An extension of the redox-responsive, transient, stiffness-controlled, cryogel matrices would involve the photochemical, light-triggered activation of the Fe^3+/2+^-CMCMA. Particularly interesting would be the design of an integrated, photosensitizer-loaded cryogel, especially photosensitizers operating in the visible (or IR) spectral region. The use of light as a trigger to transform the Fe^3+^-CMCMA cryogels to the Fe^2+^-CMCMA state is particularly interesting since light is a “non-waste” generating trigger, in contrast to ascorbate. Light as a trigger enables tunability of excitation wavelength, and modulations of intensity and duration. Moreover, application of light-activated gels for therapeutic controlled-release is particularly attractive, since laser optical devices are commonly used in therapeutics.

Nevertheless, for any practical light-stimulated cyclic activation of the cryogel, a nonleakable matrix-integrated photosensitizer should be used. Accordingly, the development of biocompatible photosensitizer-functionalized light-triggered transient release of environmentally friendly constituents (food, agricultural agents) could be a future practical application, and the use of biocompatible photosensitizer for transient release of therapeutic loads could have broad medical applications. Photosystem I (PS-I) extracted from native sources (plants, algae, or photosynthetic bacteria), exhibits biocompatibility and photoactivity in the visible light spectrum (Figure S7). Its molecular weight, 300 kDa, ensures confinement to the gel framework. PS-I was previously employed as a photosensitizer, inducing electron-transfer processes in artificial photosynthetic systems and electron-transfer cascades.^81,82^ PS-I was integrated into the Fe^3+/2+^-CMCMA cryogel. The photosensitized reduction of the Fe^3+^-CMCMA to the Fe^2+^-CMCMA (using MES as sacrificial electron donor in the buffer solution), and the concomitant transient aerobic oxidation of Fe^2+^-CMCMA to Fe^3+^-CMCMA, and the accompanying transient stiffness properties of the gel are schematically presented in Figure 5(A). Rheometry experiments probing the temporal *G*′-values of the PS-I-loaded cryogel, upon exposure of the cryogel to λ > 400 nm light source (50 mW/cm^2^) for different time intervals, under aerobic conditions, are displayed in Figure 5(B). Subjecting the cryogel to the light trigger is accompanied by a primary decrease of *G*′, reflecting the decrease of the cryogel stiffness. The Δ*G*′ values are higher as the exposure time interval to the light source increases, consistent with the higher degree of transforming Fe^3+^-CMCMA to the lower stiffness Fe^2+^-CMCMA state. Switching off the light results in a temporal transient recovery of the *G*′-values, consistent with the temporal transition of Fe^2+^-CMCMA to Fe^3+^-CMCMA. As the initial Δ*G*′ value is higher (longer exposure to the light), the transient stiffness recovery is prolonged.

**Figure 5:**
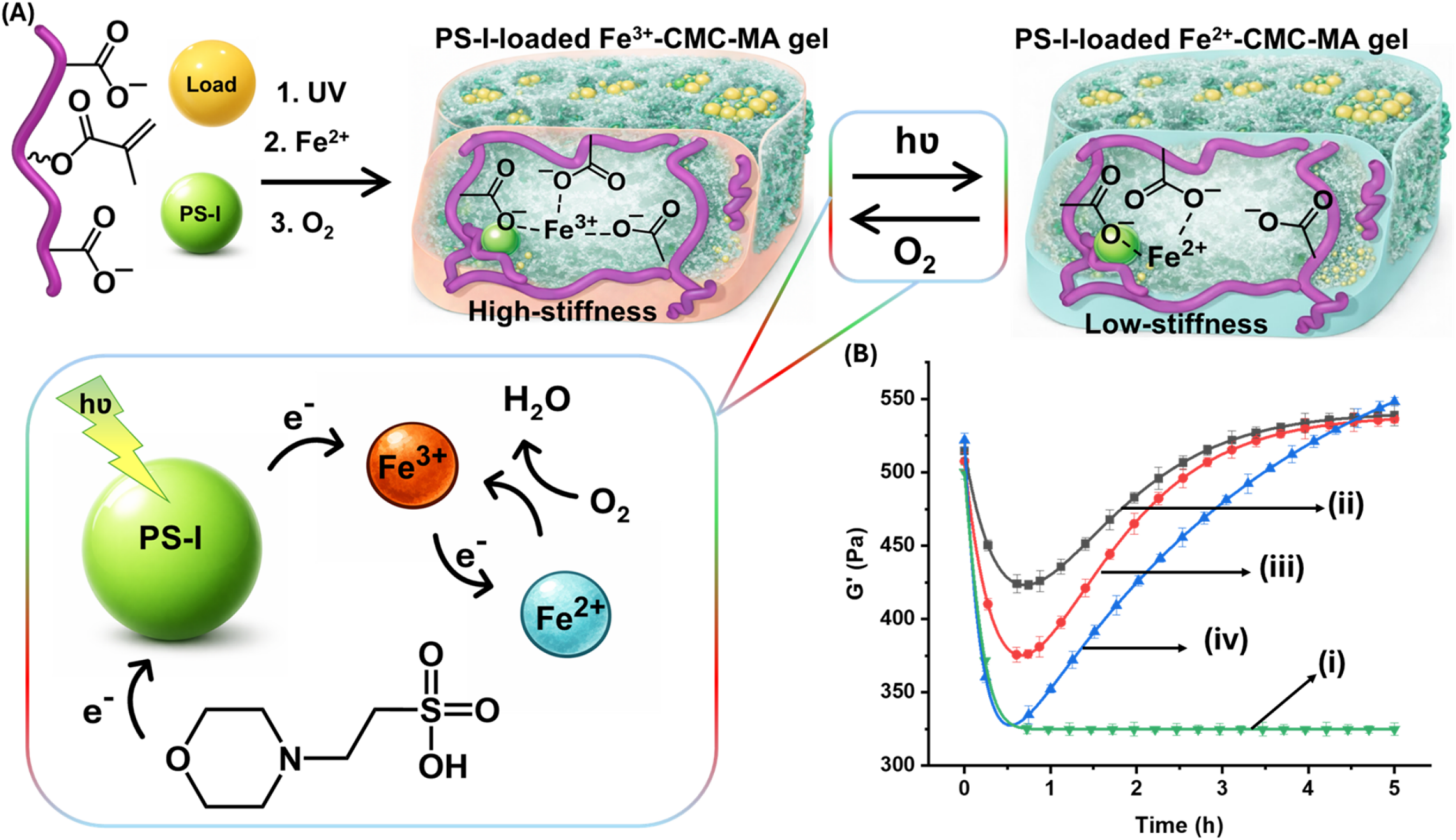
(A) Schematic synthesis of PS-I-loaded Fe^3+^-CMCMA cryogel framework and its light-triggered photosensitized electron transfer mediated transition from higher-stiffness Fe^3+^-CMCMA state to the lower-stiffness Fe^2+^-CMCMA state, undergoing, under aerobic conditions in the dark, temporal recovery to the Fe^3+^-CMCMA. (B) Rheometry measurements following the temporal G′-values upon the light-triggered transition of the Fe^3+^-CMCMA to the Fe^2+^-CMCMA state: (i) Illumination time corresponding to 40 min under N_2_ environment. Aerobic light illumination times corresponding to (ii) 20 min, (iii) 30 min, and (iv) 40 min, followed by the aerobic reoxidation transitioning from Fe^2+^-CMCMA to Fe^3+^-CMCMA.

### Light-Triggered Transient Load-Release from Fe^3+/2+^-CMCMA Cryogel Matrices

The light-triggered transient stiffness changes of the Fe^3+/2+^-CMCMA cryogel were implemented for the transient release of loads, Figure 6. The PS-I-loaded cryogel was co-loaded with tetramethyl rhodamine-modified dextran (TMR-D). While no release of TMR-D is observed in the dark, Figure 6(A), curve (i), indicating that the high-stiffness Fe^3+^-CMCMA prevents leakage of the load, subjecting the cryogel to visible light irradiation triggers the release of the TMR-D, curves (ii)–(iv). As the exposure of the cryogel to the light source is prolonged, the release of TMR-D is enhanced, consistent with the elevated stiffness decrease values of the gel at longer light-dose exposure that facilitates the load-release. After switching off the light, the load-release processes are temporarily slowed down and reach saturation values. The saturation values are due to the temporal aerobic oxidation of the Fe^2+^-CMCMA cryogel to the high-stiffness Fe^3+^-CMCMA framework that prohibits the load-release processes. Figure 6(B) depicts the temporal release rates of the loads (time-dependent derivative of the load-release profiles depicted in Figure 6(A)). Evidently, the release rates demonstrate dissipative, transient patterns where the light-triggered transition of the Fe^3+^-CMCMA to the Fe^2+^-CMCMA state leads to primary peak release rates, which temporarily slow down to the parent “locked” release state. Figure 6(C) depicts the cyclic transient light-induced release of the load, TMR-D, upon repeated light/O_2_ cycles. Furthermore, Figure 6(D) demonstrates the light-triggered release rates of the load using two transient release cycles. The transient depletion of the release of TMR-D in the first cycle is followed by the light-triggered formation of the lower stiffness Fe^2+^-CMCMA gel that results in the second transient release cycle.

**Figure 6:**
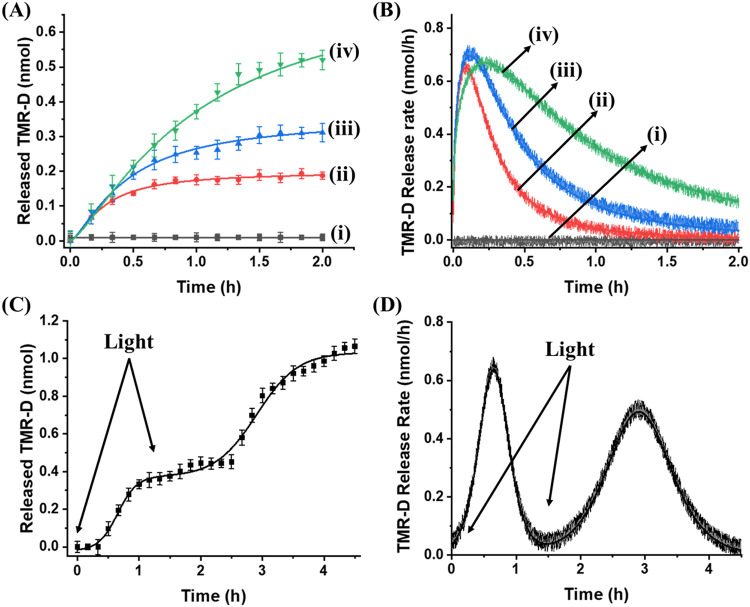
(A) Time-dependent light-triggered release of TMR-D from the PS-I-loaded Fe^3+^-CMCMA gel, irradiated under aerobic conditions for different time intervals: (i) no light irradiation, (ii) 20 min, (iii) 30 min, and (iv) 40 min. (B) Transient release rates of TMR-D from the Fe^3+^-CMCMA corresponding to the time-dependent derivative of curves (i–iv) presented in (A). (C) Cyclic time-dependent light-induced release of TMR-D from the Fe^3+^-CMCMA under aerobic dark conditions (time interval of illumination 30 min). (D) Cyclic transient release rate of TMR-D from the Fe^3+^-CMCMA matrix corresponding to the time-dependent derivative presented in (C).

The light-triggered release of insulin, or the anti-VEGF aptamer, and the transient release rates are displayed in Figure 7(A),(B). The phototriggered release of the anti-VEGF aptamer is noteworthy due to the possibility of adapting these systems for ocular therapeutic applications. Anti-VEGF aptamer inhibits VEGF, a major constituent in the angiogenesis pathway (formation of blood vessels). The retina-enhanced VEGF-induced formation of blood vessels is a major cause of blindness in diabetic patients, and aptamer-stimulated inhibition of the process has been supported as a possible therapy. The use of photoresponsive Cy3-modified anti-VEGF aptamer-loaded PS-I-functionalized Fe^3+^-CMCMA microgels could provide cyclic dose-controlled release of the therapeutic agent.

**Figure 7:**
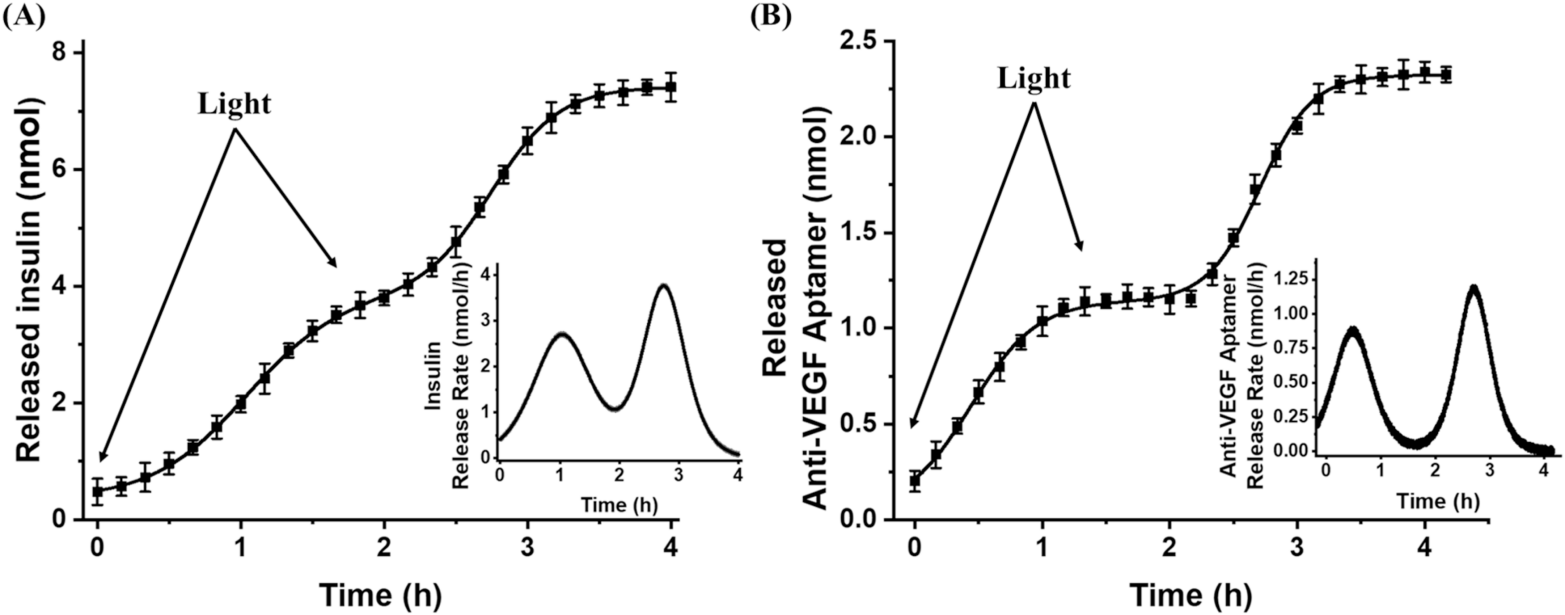
(A) Cyclic temporal light-triggered release profile of fluorophore-labeled insulin under aerobic conditions upon the triggered photosensitized electron transfer-mediated transition from Fe^3+^-CMCMA to Fe^2+^-CMCMA, and back to the oxidized Fe^3+^-CMCMA. Inset: Cyclic release rate of the labeled insulin (time-dependent derivative of the release profile). (B) Cyclic temporal light-induced release profile of fluorophore-labeled anti-VEGF aptamer under aerobic conditions upon the triggered redox-mediated dissipative stiffness process. Inset: Cyclic release rate of the labeled anti-VEGF aptamer (time-dependent derivative of the release profile).

It should be noted that the release of the different payloads from the different gel frameworks could be transiently switched for eight cycles. Nevertheless, the content and rate of the released loads decreased progressively upon cycling due to the decrease of the load concentrations in the gel matrices. Nevertheless, the content/release rates of the loads could be adjusted to comparable values by adjusting and modulating the dose of the release trigger (concentration of ascorbate or time-intervals of illumination) and by reducing the number of release cycles. Moreover, we note that the frameworks cannot be reloaded after releasing the payloads.

### Light-Triggered Self-Healing of PS-I-Loaded Fe^3+/2+^-CMCMA Cryogel Matrices

In addition, light-induced self-healing of PS-I-functionalized Fe^3+^-CMCMA cryogels was accomplished. Figure 8(A), Panel I depicts the PS-I-loaded Fe^3+^-CMCMA (a) that was irradiated by visible light to transform to the low-stiffness Fe^2+^-CMCMA state (b), then cut into two pieces (c) that were further physically connected under aerobic conditions (d). The physically connected pieces underwent self-healing into an integrated gel matrix (e) and (f), while the healing process of the physically connected pieces under anaerobic conditions was prohibited, Figure 8(A), Panel II, images (g–l). The self-healing of the Fe^3+^-CMCMA gel under aerobic conditions is attributed to the aerobic-induced formation of enhanced cross-linking at the interconnecting boundary from the Fe^2+^-CMCMA to the Fe^3+^-CMCMA state, which led to the healing process. Indeed, “dog-bone” self-healing experiment, Figure 8(B), Panel I­(a–e), was conducted, and stress/strain stretching experiments, Figure 8(B), Panel II, demonstrated that the Young’s modulus of the parent PS-I-loaded Fe^3+^-CMCMA cryogel corresponds to 5 kPa, curve (i), while light-triggered transition to the Fe^2+^-CMCMA state yielded a substantially softer gel (Young’s modulus = 1 kPa), curve (ii). The aerobically recovered healed cryogel revealed a significantly higher Young’s modulus, curve (iii), close to the parent Fe^3+^-CMCMA state.

**Figure 8:**
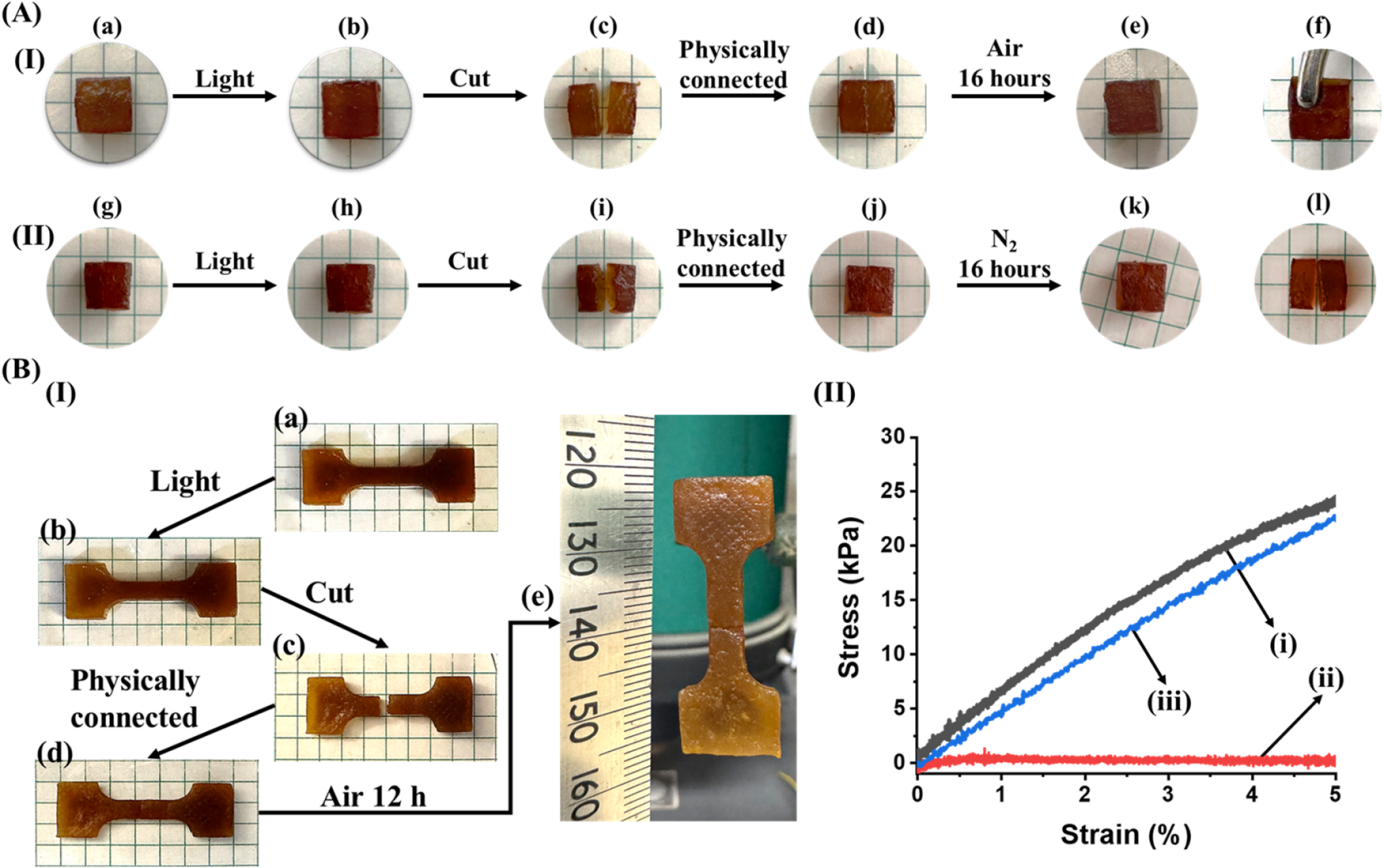
(A) Self-healing of the light-triggered Fe^3+/2+^-CMCMA gel, and corresponding visual images of: Panel I–the stepwise self-healing of two Fe^2+^-CMCMA pieces, physically interacted under aerobic conditions in the dark. Panel II–Control system where the physically connected light-treated pieces are left under N_2_in dark conditions, prohibiting their self-healing process. (B) Quantitative evaluation of the quality of the self-healing process by monitoring the strain/stress stretching: Panel I–structure of the gel device probing the stress/strain parameters upon the light-induced self-healing process of the Fe^2+^-CMCMA physically connected subunits, under aerobic conditions in dark. Panel II–Stress/strain curves corresponding to (i)—The PS-I-loaded Fe^3+^-CMCMA gel device, (ii)—light-generated Fe^2+^-CMCMA gel device prior to cutting, and (iii)—the cut Fe^2+^-CMCMA device, physically connected and allowed to self-heal under aerobic conditions for 12 h in the dark.

### Thermal and Ascorbate/Light-Triggered Soft-Robotic Functions of pNIPAM/Fe^3+/2+^-CMCMA Bilayer Cryogel Devices

The ascorbate/light-triggered transient stiffness properties of the Fe^3+/2+^-CMCMA cryogels were then implemented to develop bimodal thermal/ascorbate or thermal/light-driven soft robotic bilayer bending devices. Figure 9(A) depicts schematically the composition of the bilayer device and the principles of the mechano-robotic operation of the system. A thermoresponsive pNIPAM cryogel layer (40 mm length × 4 mm width × 2 mm height dimensions) was prepared in a Teflon mold, and a second layer comprised of the Fe^3+^-CMCMA cryogel (40 mm length × 4 mm width × 5 mm height dimensions) was polymerized on top of the primer pNIPAM layer. The extruded bilayer system and the respective dimensions of the device resulted in, at 25 °C, a bilayer configuration of comparable stiffnesses, reflected by the almost linear shape of the device, Figure 9(B), Panel I (a). In this experiment, the upper, thicker, colored layer corresponds to the Fe^3+^-CMCMA gel, and the transparent layer below corresponds to the pNIPAM layer. Heating of the device from 25 to 38 °C is anticipated to induce the pNIPAM gel-to-solid transition. This phase transition leads to stress-differing-induced bending of the bilayer device into a curved configuration that follows the shrunk structure of the pNIPAM. This is evident by the visual formation of a turbid white layer of heated, solid layer of pNIPAM, and the mechanical stress-induced bending of the device into a curved structure (diameter ca. 29.4 mm) dictated by the high rigidity of the pNIPAM layer and the stiffness state of the Fe^3+^-CMCMA layer, Panel I (b). The thermal-induced bending process proceeds within 12 min, and after this time interval, the geometrical features of the device remained constant, Panel I (c). Figure 9(B), Panel II depicts the ascorbate-induced enhanced bending of the bilayer device. After the primary thermal-induced bending of the bilayer device reaches an equilibrated curved configuration, subjecting the curved bilayer configuration to ascorbate at 38 °C for 10 min results in the reduction of the Fe^3+^-CMCMA layer to the lower stiffness Fe^2+^-CMCMA. This results in a further enhanced bending of the curved bilayer structure, Panel II (d, e), into a curved, worm-like configuration, consistent with the increased stiffness differences between the pNIPAM/Fe^2+^-CMCMA layers, and the accompanying stress interactions present in the bilayer device. Transferring the pNIPAM/Fe^2+^-CMCMA bent device to an ascorbate-free solution at 38 °C induces the transient transition of the device toward the opposite direction. The curved pNIPAM/Fe^2+^-CMCMA bilayer structure undergoes, at 38 °C, a slow aerobic oxidation to the curved pNIPAM/Fe^3+^-CMCMA state, Figure 9 (B), Panel III (f–h), that upon cooling the system to 25 °C recovers into the parent linear bilayer configuration, Figure 9 (B), Panel IV (i–k). It should be noted that although the reduction of the Fe^3+^-CMCMA layer to the Fe^2+^-CMCMA is relatively fast, the accompanying adaptive stiffness and stress-difference interactions within the bilayer device, which lead to the reversed, open, temporal topological structures, are substantially slower. The mechanomorphing thermal/ascorbate transitions shown in Figure 9 (B) are reversible and were reproduced for four cycles with no noticeable operative perturbations. Evaluation of the curvature (1/*r*) was performed using eq 1.

1 $$r=\frac{y^{2}}{8x}+\frac{x}{2}$$

**Figure 9:**
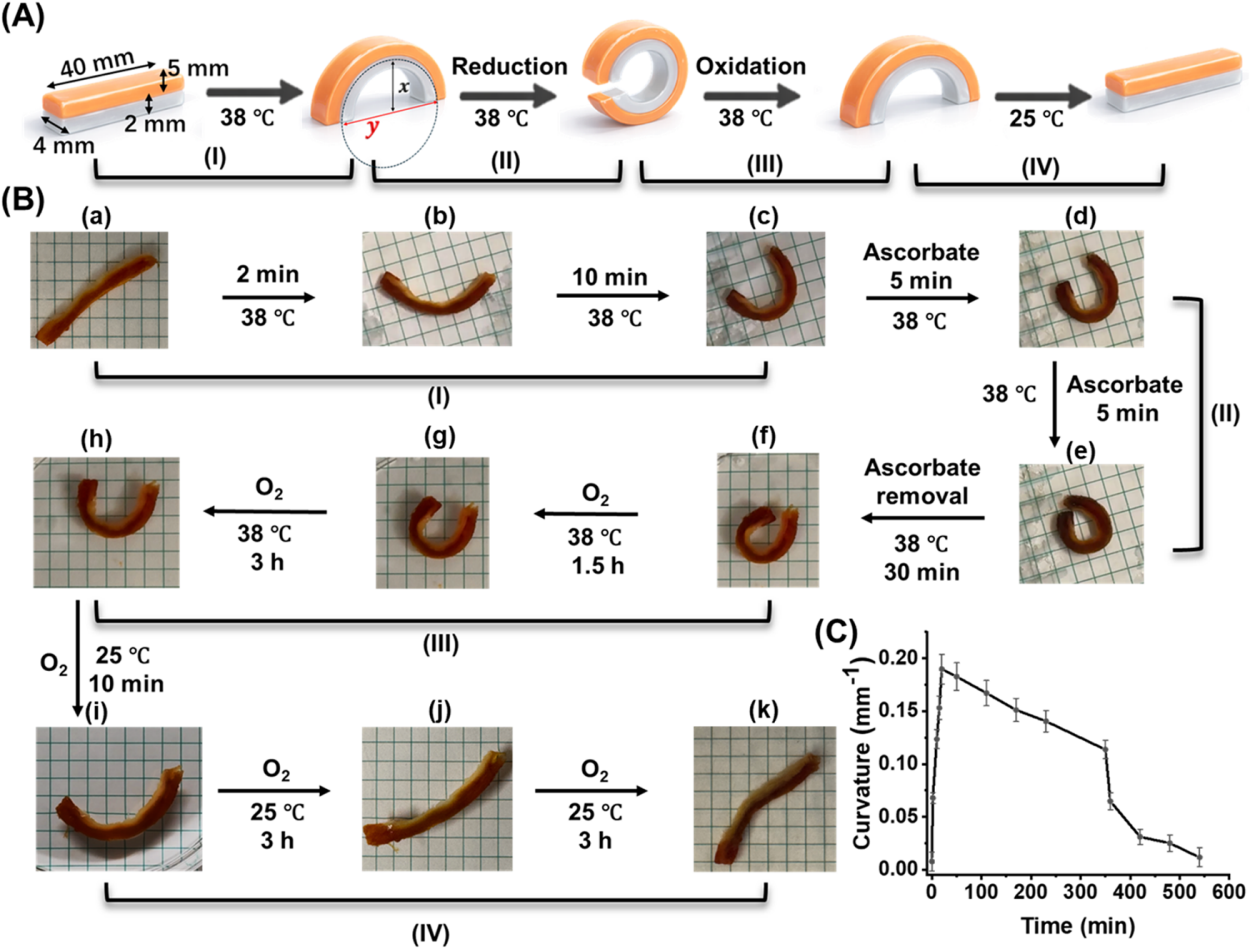
(A) Schematic temporal dynamic bending of a bilayer device consisting of an ascorbate-triggered Fe^3+^-CMCMA cryogel layer (brown/orange) and a thermoresponsive pNIPAM layer cryogel (transparent/gray). Thermal-induced gel-to-solid phase transition leads to a stress difference between the layers, resulting in bending of the device forward toward the pNIPAM layer, whereas ascorbate-induced reduction of Fe^3+^-CMCMA to the lower-stiffness Fe^2+^-CMCMA gel enhances the stress difference between the two layers, resulting in further bending of the device toward the pNIPAM layer, yielding a circular “worm-like” framework. Aerobic reoxidation of the structure to the Fe^3+^-CMCMA layer, decreases the stress difference between the layers, leading to the temporal opening of the circular framework. The subsequent cooling of the system results in a solid-to-gel phase transition, resulting in the parent linear bilayer device. (B) Images following the temporal mechanomorphing features of the device: Panel I—Temporal temperature-induced bending of the bilayer device mediated by the thermoresponsive pNIPAM gel-to-solid phase transition. Panel II—Ascorbate-triggered reduction of Fe^3+^-CMCMA to the lower-stiffness Fe^2+^-CMCMA enhances the stress difference between the layers, accompanied by further temporal bending actuation of the device into a circular topology. Panel III—Aerobic reoxidation of the Fe^2+^-CMCMA layer to the higher-stiffness Fe^3+^-CMCMA gel state decreases the stress difference between the layers, resulting in the temporal opening of the circular framework. Panel IV—Cooling the framework to 25 °C leads to pNIPAM solid-to-gel phase transition and to the reconfiguration of the device into the parent linear topology. (C) Transient curvature corresponding to the different states of the device.

In this equation, *r* is the radius of the bent configuration, y stands for the distance separating the ends of the curved structure, and *x* is the maximum height of the curved structure. Figure 9(C) depicts the time-dependent temporal curvature of the bilayer gel device undergoing the heat-induced, ascorbate-enhanced bending process.

Similarly, thermal/light-induced controlled bending is demonstrated in Figure 10(A). A bilayer gel device consisting of thermoresponsive pNIPAM cryogel layer (40 mm length × 4 mm width × 2 mm height dimensions) coupled to a photoresponsive PS-I-loaded Fe^3+^-CMCMA cryogel layer (40 mm length × 4 mm width × 5 mm height dimensions) was assembled in a Teflon mold, yielding, at 25 °C, an almost linear bilayer device, Panel I (a). This suggests that no significant stress differences between the two layers exist. Heating the bilayer assembly to 38 °C results in the gel-to-solid phase transition of the pNIPAM layer, resulting in the bending of the device due to the high-stiffness of the heated pNIPAM layer and the stress difference between the two layers developed in the system, Panel I (b, c). The cooling of the system to 25 °C recovered the almost linear configuration, due to the reversed solid-to-gel phase transition of the pNIPAM cryogel layer. The light-induced PS-I photosensitized electron-transfer reduction of the Fe^3+^-CMCMA layer (illumination at λ > 400 nm, *P* = 50 mW/cm^2^) for 30 min in a MES buffer solution of the thermally induced bent device at 38 °C resulted in the formation of a lower-stiffness Fe^2+^-CMCMA layer. Formation of the Fe^2+^-CMCMA enhanced the stress difference between the two layers, leading to further bending of the device, Panel II (d, e). The subsequent aerobic oxidation of the Fe^2+^-CMCMA layer to the Fe^3+^-CMCMA state, Panel III (f–h), and the cooling of the system to 25 °C restore the primary, almost linear configuration of the bilayer device, Panel IV­(i–k). Figure 10(B) depicts the temporal dynamic mechanomorphed curvature of the bilayer PS-I-loaded pNIPAM/Fe^3+^-CMCMA device upon the thermally/light-triggered controlled stiffness changes of the layers and the accompanying aerobic oxidation of the Fe^2+^-CMCMA layer under dark conditions. For images and time-dependent curvature of a bilayer device during temperature-induced bending processes, see Figure S8.

**Figure 10:**
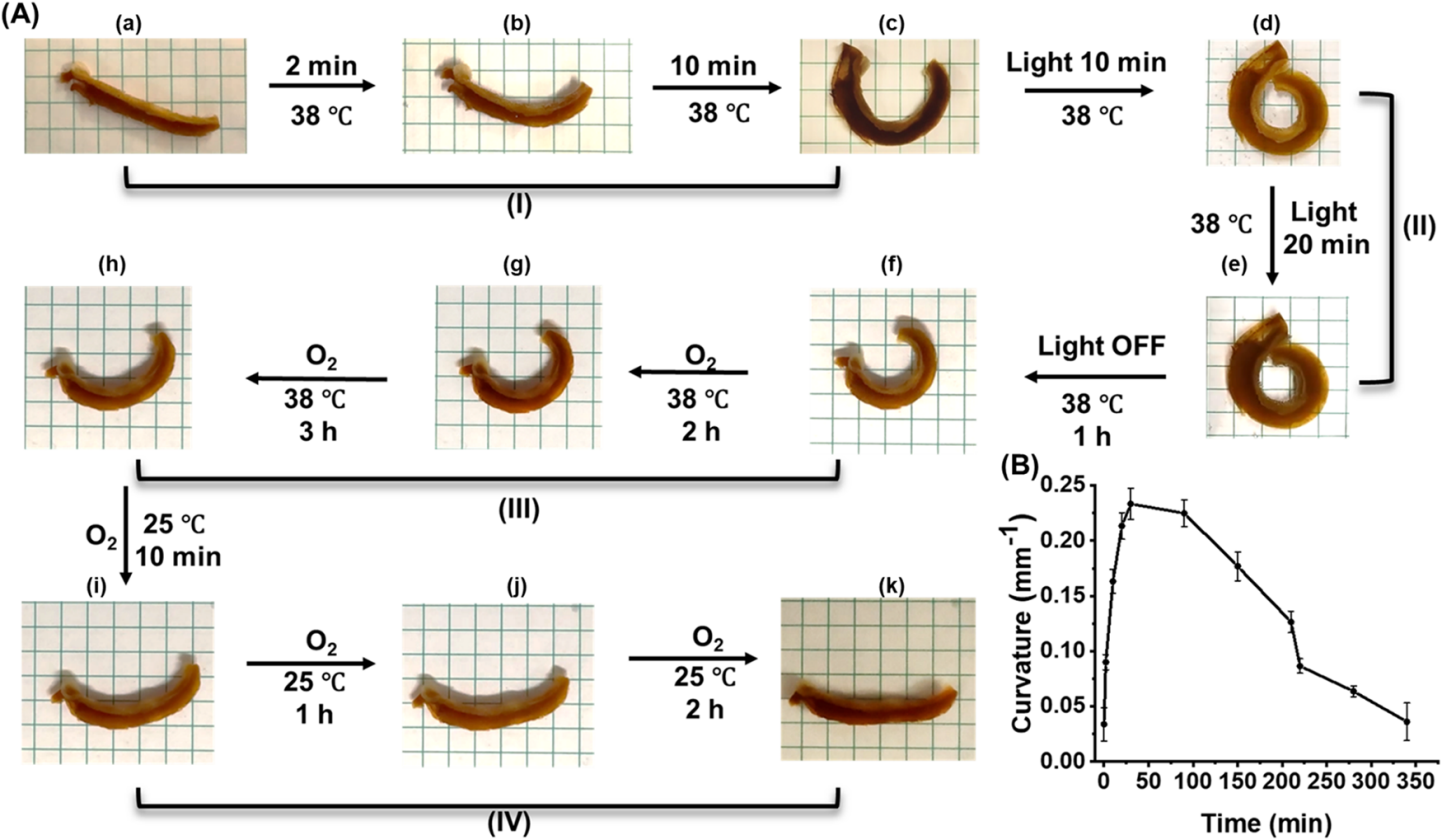
(A) Images corresponding to the stepwise temporal mechanomorphing features of the PS-I-loaded mechanomorphing device: Panel I—Elevated temperature-induced temporal bending of the device mediated by the thermoresponsive pNIPAM gel-to-solid phase transition. Panel II—Light-mediated photosensitized reduction of Fe^3+^-CMCMA to the lower-stiffness Fe^2+^-CMCMA enhances the stress difference between the layers, accompanied by further temporal bending actuation of the device into a circular topology. Panel III—Aerobic reoxidation in the dark of the Fe^2+^-CMCMA layer to the higher-stiffness Fe^3+^-CMCMA gels state decreases the stress difference between the layers, resulting in the temporal opening of the circular framework. Panel IV—Cooling to 25 °C leads to the pNIPAM solid-to-gel phase transition and to the reconfiguration of the device into the parent linear topology. (B) Transient curvature transitions during the thermal/light-induced activation of the device.

## Conclusions

The study introduced Fe^3+/2+^-carboxylate-cross-linked carboxymethyl cellulose methacrylate, Fe^3+/2+^-CMCMA, cryogels as redox-responsive functional matrices, revealing transient stiffness properties. By chemical reduction of Fe^3+^-CMCMA matrix to Fe^2+^-CMCMA, or light-induced photosensitized electron-transfer reduction of Fe^3+^-CMCMA framework to Fe^2+^-CMCMA using the photosynthetic reaction center, PS-I, as a photosensitizer loaded in the gel, the triggered switching of the gel stiffness from higher stiffness to lower stiffness was demonstrated. The subsequent aerobic oxidation of the Fe^2+^-CMCMA (lower stiffness state) to the Fe^3+^-CMCMA (higher stiffness state) led to functional gel matrices, revealing chemical/light-triggered transient stiffness properties under aerobic conditions. The transient, dissipative stiffness properties of the gels were characterized by rheometry and stress/strain stretching measurements, and complementary structural microscopy evaluations. Moreover, the transient Fe^3+^/Fe^2+^ oxidation states accompanying the transient dissipative processes were supported by XPS. The switchable transient stiffness properties of the gels were implemented to enable switchable, cyclic, transient, dose-controlled release of loads, including tetramethyl rhodamine-dextran, insulin, or the anti-VEGF aptamer. Moreover, the switchable stiffness functions of the gel and the aerobic reoxidation of the Fe^2+^-CMCMA state to the Fe^3+^-CMCMA state were applied to use the stimuli-responsive gel as a dynamic self-healing matrix, demonstrating the potential use of the gel for regenerative medicine. Also, the transient stiffness properties of the Fe^3+/2+^-CMCMA gel were implemented to assemble pNIPAM/Fe^3+/2+^-CMCMA bilayer systems and PNIPAM/PS-I-loaded-Fe^3+/2+^-CMCMA bilayer devices for the thermal/chemical or thermal/photochemical bimodal-triggered, transient operation mechanomorphing of a soft robotic actuation bending system.

The significance of the present study rests, however, on the versatility of the research results and the emerging challenges for future exploration and practical applications. The results suggest that many other acrylate-modified carboxylate polysaccharides, such as alginate or hyaluronic acid, could be polymerized into gel frameworks and further cross-linked by Fe^3+^-carboxylate frameworks, yielding redox-responsive gels exhibiting cyclic transient switchable stiffness properties. The triggered control of the transient stiffness functions of the gels by ascorbate (vitamin C) or the native photosynthetic PS-I is particularly important for future biomedical applications. Specifically, the controlled, transient, dose-controlled release of the anti-VEGF aptamer or of insulin from the stimuli-responsive gels supports the potential for combating ocular diseases,^83–85^ such as angiogenesis-induced age-related macular degeneration (**AMD**)^86,87^ or noninvasive administration of insulin delivery.^88,89^

The present study emphasized the redox-responsive, transient stiffness properties of the Fe^3+^-CMCMA gels as bulk matrices. The miniaturization of the frameworks into microgels or microcapsules^90–92^ is anticipated to introduce new dimensions to their biomedical applications. Furthermore, the assembly of the gels on surfaces, particularly conductive supports, would enable the electrochemical triggering of the gels by electrical inputs for controlled release of loads.^93–95^ Moreover, the patterning of surfaces with stimuli-responsive redox-active gel substrates and their controlled stiffness could control sequential interactions with cells, reflected by controlled cell patterning,^96^ cell migration,^97–99^ proliferation,^100,101^ or their maturation to differentiated cells.^102–106^

## Supplementary Material



## Data Availability

The data that support the findings of this study are available from the corresponding author upon reasonable request.

## Notes

Methods for the different gel fabrications and characterizations are presented. These include: SEM imaging, XPS analysis, rheometry and tensiometry, preparation of fluorophore-conjugated loads and load-release quantifications, and analysis of bilayer mechanomorphing curvature (DOCX)
