# Supplementary material for: Redox- and Photo-Responsive Fe3+/2+-Cross-Linked Carboxymethyl Cellulose Methacrylate Dissipative Gels: Synthesis and Applications
Source: ACS Appl Mater Interfaces. 2026 Jun 16;18(25):35059–73. doi: 10.1021/acsami.6c07915 (PMC13339012; doi:10.1021/acsami.6c07915)
Supplement: Supplementary file 1 [file am6c07915_si_001.docx]

**Supporting Information**

Redox-and Photo-Responsive Fe^3+/2+^-Crosslinked Carboxymethyl Cellulose Methacrylate Dissipative Gels: Synthesis and Applications *Jianghe Zhao^1^, Yunlong Qin^1^,* *Muhammad Abdel-Haq^2^, Vitaly Gutkin^3^, Ehud Neumann^4^, Abraham J. Domb^2^,* *Rachel Nechushtai^4^, Yuwei Hu^5^, Junji Zhang^6^, Gilad Davidson-Rozenfeld^1,7*^, and Itamar Willner^1,3*^*

^1^Institute of Chemistry, The Center for Nanoscience and Nanotechnology, The Hebrew University of Jerusalem, Jerusalem 91904, Israel

E-mail: [itamar.willner@mail.huji.ac.il](mailto:itamar.willner@mail.huji.ac.il)

E-mail: [gilad.davidson@mail.huji.ac.il](mailto:gilad.davidson@mail.huji.ac.il)

^2^Faculty of Medicine, School of Pharmacy, The Hebrew University of Jerusalem, Jerusalem 9112002, Israel. E-mail: [avid@ekmd.huji.ac.il](mailto:avid@ekmd.huji.ac.il).

^3^The Harvey M. Krueger Family Center for Nanoscience and Nanotechnology Edmond J. Safra Campus. The Hebrew University of Jerusalem, Jerusalem 9190401, Israel.

E-mail: [vitalyg@savion.huji.ac.il](mailto:vitalyg@savion.huji.ac.il)

^4^The Alexander Silberman Institute of Life Sciences, The Hebrew University of Jerusalem, Jerusalem 9190401, Israel

E-mail: [rachel@mail.huji.ac.il](mailto:rachel@mail.huji.ac.il)

^5^School of Chemical Engineering and Technology, Sun Yat-Sen University, Zhuhai, Guangdong 519082, P. R. China.

Email: [yuweihu85@gmail.com](mailto:yuweihu85@gmail.com)

^6^Key Laboratory for Advanced Materials and Joint International Research Laboratory of Precision Chemistry and Molecular Engineering, Feringa Nobel Prize Scientist Joint Research Center, Frontiers Science Center for Materiobiology and Dynamic Chemistry, Institute of Fine Chemicals, School of Chemistry and Molecular Engineering, East China University of Science and Technology, Shanghai 200237, China

^7^Food Science Department, Tel-Hai Academic College, D.N. Upper Galilee, Kiryat Shmona 12210, Israel

E-mail: [Giladdav@m.telhai.ac.il](mailto:Giladdav@m.telhai.ac.il)

**Table of Contents**

[**1. Experimental section**](#_Toc220566472)**...................................................................................................S6**

[**1.1 Reagents and materials**](#_Toc220566473)**.............................................................................................S6**

[**1.2 Instrumentation**](#_Toc220566476)**.........................................................................................................S6**

[**1.3 Methods**](#_Toc220566478)**......................................................................................................................S7**

[**1.3.1 Gel fabrication**](#_Toc220566479)**........................................................................................................S7**

[**1.3.2 SEM sample preparation of Fe³⁺-CMCMA cryogels and hydrogels**](#_Toc220566486)**..................S9**

[**1.3.3 XPS-Based Quantification of Fe³⁺/Fe²⁺ Ratio in the gel.......................................S9**](#_Toc220566488)

[**1.3.4 Chemical-driven transient, dissipative stiffness properties of Fe³⁺-CMCMA cryogel**](#_Toc220566489)**.............................................................................................................................S10**

[**1.3.5 Chemical-driven transient, dissipative tensile properties of Fe³⁺-CMCMA cryogel**](#_Toc220566491)**.............................................................................................................................S10**

[**1.3.6 Photochemical-driven transient, dissipative stiffness properties of PS-I-loaded Fe³⁺-CMCMA cryogel**](#_Toc220566493)**....................................................................................................S10**

[**1.3.7 Photochemical-driven transient, dissipative tensile properties of PS-I-loaded Fe³⁺-CMCMA cryogels**](#_Toc220566495)**..................................................................................................S11**

[**1.3.8 Calculation of Young’s modulus from tensile stress-strain curves**](#_Toc220566497)**..................S11**

[**1.3.9 Preparation of insulin-FITC conjugates**](#_Toc220566497)**.............................................................S12**

[**1.3.10 Loading of Fe³⁺-CMCMA cryogels with fluorophore-labeled conjugates**](#_Toc220566499)**.....S12**

[**1.3.11 Chemical-driven transient, dissipative load release**](#_Toc220566501)**........................................S13**

[**1.3.12 Photochemical-driven transient, dissipative load release**](#_Toc220566502)**...............................S13**

[**1.3.13 Bilayer device fabrication**](#_Toc220566503)**..................................................................................S14**

[**1.3.14 Chemical-driven bending of the bilayer device**](#_Toc220566504)**................................................S14**

[**1.3.15 Photochemical-driven bending of the bilayer device**](#_Toc220566506)**.......................................S15**

[**2. Rheometry characterization of Fe³⁺/Fe^2+^-CMCMA cryogels and hydrogels before and after reduction**](#_Toc220566508)**........................................................................................................S15**

[**3. XPS analysis: quantification of Fe³⁺/Fe²⁺ ratio in transient, dissipative Fe³⁺-CMCMA cryogel**](#_Toc220566514)**, for a detailed analysis of the Fe³⁺/Fe²⁺ ratio and the deconvolution process implemented to quantify the transient Fe³⁺/Fe²⁺ ratio.................................................................................................................................S18**

**4. Calibration curves for fluorophore-labeled conjugates.........................................S20**

[**5. Quantification of the fluorophore-labeled conjugates ratio**](#_Toc220566522)**..................................S21**

[**6. UV-vis absorption spectra of PS-I**](#_Toc220566522)**............................................................................S24**

[**7.**](#_Toc220566506) **Bending Of Bilayer Devices and the Transient Curvature Characterization…..S25**

[**8. Reference**](#_Toc220566528)**....................................................................................................................S26**

1. **Experimental section**

**1.1 Reagents and materials****.**

All reagents were purchased from commercial suppliers and used without further purification. Carboxymethyl cellulose glycidyl methacrylate (CMCMA, degree of methacrylation 10%), iron(II) sulfate heptahydrate (FeSO₄·7H₂O), L-ascorbic acid, lysine fixable tetramethlrhodamine dextran, 70,000 MW (TMR-D), insulin, fluorescein isothiocyanate isomer I (FITC), 2-(N-morpholino)ethanesulfonic acid (MES), Magnesium sulfate (MgSO₄), glycerol, Calcium chloride (CaCl_2_), n-Dodecyl-β-D-maltoside (DDM), sodium hydroxide, N-isopropylacrylamide (NIPAM), N,N,N’,N’-tetramethylethylenediamine (TEMED), and ammonium persulfate (APS, >98%) were purchased from Sigma Aldrich Co. (St. Louis, MO). Photosystem I (PS-I) was isolated in the laboratory of Prof. Rachel Nechushtai as previously described in detail.^1^ DNA strands were purchased from Integrated DNA Technologies Inc. (IDT). All oligonucleotides were supplied as HPLC-purified aqueous solutions (100 nM) by the supplier. The sequences of the oligonucleotides used in this study are listed below.

Anti-VEGF aptamer (5’-Cy3-labeled): 5’-Cy3-TGTGGGGTGGACGGGCCGGGTAGA-3’.

**1.2 Instrumentation**

Ultrapure deionized water from NANOpure Diamond (Barnstead) was used in all experiments. Scanning electron microscopy (SEM) images were acquired using a Thermo Scientific Apreo 2-S scanning electron microscope (Thermo Fisher Scientific). Tensile tests were performed using an LS1^+^ universal testing machine (AMETEK Lloyd Instruments, USA). Ultraviolet-visible (UV-vis) absorption spectra were recorded with a temperature-controlled UV-2401PC spectrophotometer (Shimadzu, Japan). Time-dependent G’/G’’ (Pa) values were measured by a HAAKE MARS III rheometer (Thermo Scientific). Photochemical experiments were carried out using a continuous-mode xenon lamp system (Hamamatsu Photonics, model E7536, Japan). Fluorescence spectra were collected with a Cary Eclipse Fluorometer (Varian Inc.), using a quartz cuvette of 1 cm path length. X-ray photoelectron spectroscopy (XPS) measurements were performed using a Kratos AXIS Supra spectrometer (Kratos Analytical Ltd., Manchester, U.K.) with an Al Kα monochromatic radiation X-ray source (1486.6 eV). XPS samples were prepared in a Glovebox system (Vigor, SG series, China) and transferred by an Air Sensitive Transporter (Kratos Analytical Ltd.).

**1.3 Methods**

**1.3.1 Gel fabrication**

**Fe³⁺-CMCMA cryogel fabrication****.** A CMCMA solution (2 % w/w) containing APS (16.6 mM) was transferred into a round plastic mold (diameter 10 mm). After nitrogen degassing for 5 min, TEMED (33 mM) was added, yielding a final total volume of 120 μL. Then, the sample was immediately placed at -20 °C for 4 h. The resulting cryopolymerized matrix was then immersed in 20 mL of an aqueous Fe²⁺ solution (20-40 mM) prepared in MES buffer (pH 7.2) and sealed under an anaerobic atmosphere for 24 h to allow sufficient coordination of iron ions with the carboxylate groups of CMCMA. Then, the samples were incubated under ambient conditions for 3 days, allowing iron penetration and spontaneous oxidation and formation of iron-crosslinked cryogels. For tensile testing, the same precursor solution (2 mL) was cast into a custom dog-bone-shaped mold with a gauge length of 20 mm and a rectangular cross section (6 × 6 mm²), degassed with nitrogen for 10 min, mixed with TEMED, cryopolymerized at -20 °C, and subsequently incubated in Fe²⁺ solution under identical conditions as for the gels prepared in the round mold. All gels were washed and stored in MES buffer until further use.

**Fe^3+^-CMCMA hydrogel fabrication****.** A CMCMA solution (2% w/w) containing APS (16.6 mM) was transferred into a round plastic mold (diameter 10 mm). After nitrogen degassing for 5 min, TEMED (33 mM) was added, yielding a final total volume of 120 μL. Then, the sample was placed at 4 °C for 4 h to allow hydrogel formation. The resulting hydrogel was then immersed in 20 mL of an aqueous Fe²⁺ solution (20-40 mM) prepared in MES buffer (pH 7.2) and sealed under an anaerobic atmosphere for 24 h to allow sufficient coordination of iron ions with the carboxylate groups of CMCMA. Then, the samples were incubated under ambient conditions for 3 days, allowing iron penetration and spontaneous oxidation and formation of iron-crosslinked hydrogels. Hydrogels were washed and stored in MES buffer until further use.

**PS-I-loaded Fe^3+^-CMCMA cryogel fabrication****.** A CMCMA solution (2% w/w) containing APS (16.6 mM) and PS-I (0.3 mg mL^-^¹) was prepared using a PS-I stabilization buffer (12 mM MgSO₄, 40 mM MES, pH 6.0, 5% glycerol, and 0.02% DDM). The solution was transferred into a round plastic mold (diameter 10 mm), degassed with nitrogen for 5 min, and TEMED (33 mM) was added, yielding a final total volume of 120 μL. The sample was immediately placed at -20 °C for 4 h. The resulting cryopolymer was then immersed in 20 mL of an aqueous Fe²⁺ solution (20-40 mM) prepared in 10 mM MES buffer (pH 7.2) and sealed under an anaerobic atmosphere for 24 h to allow sufficient coordination of iron ions with the carboxylate groups of CMCMA. Then, the samples were incubated under ambient conditions for 3 days to allow spontaneous oxidation and formation of iron-crosslinked cryogels. For tensile testing, the same precursor solution (2 mL) was cast into a custom dog-bone-shaped mold with a gauge length of 20 mm and a rectangular cross section (6 × 6 mm²), degassed with nitrogen for 10 min, mixed with TEMED, cryopolymerized at -20 °C, and subsequently incubated in Fe²⁺ solution under identical conditions. The gels were washed and stored in buffer (12 mM MgSO₄, 40 mM MES, pH 6.0, 5% glycerol, 20 mM CaCl_2_, and 0.02% DDM) until further use.

**1.3.2 SEM sample preparation of Fe³⁺-CMCMA cryogels and hydrogels****.**

For SEM analysis, Fe³⁺-CMCMA cryogels and hydrogels were first thoroughly washed with deionized water to remove residual salts. The samples were then cut into small pieces to expose the internal structure, followed by freezing at -80 °C and lyophilization until complete dehydration. The dried gels were mounted onto aluminum stubs using conductive carbon tape. Prior to imaging, the samples were sputter-coated with a thin layer of iridium (Ir) to prevent charging.

**1.3.3 XPS-based quantification of Fe^3+^/Fe^2+^ ratio in the gel**.

The transient Fe³⁺/Fe²⁺ ratio in the gels was analyzed by XPS. Gel samples were cut into pieces, and the reduction was performed by incubating the gels in 20 mM ascorbic acid in MES buffer (pH 7.2) for 5 min, followed by three rinses with the same buffer. The samples were mounted on silicon wafers, purged with argon for 30 min, dried, and transferred to the XPS chamber using an air-sensitive transporter under an argon atmosphere in a glovebox. For XPS analysis, spectra were acquired at a takeoff angle of 90° (normal to the analyzer); the chamber vacuum was 2 × 10^-9^ torr. High-resolution XPS spectra of Fe 2p were measured with a pass energy of 20 and a 0.1 eV step size. The binding energies were calibrated using the C 1s peak energy of 285.0 eV. Data was collected and analyzed by using the ESCApe processing program and CasaXPS. For time-dependent reoxidation, reduced gels were exposed to ambient air for 0, 1, 2, 6, 8, 10, or 12 h prior to argon purging, drying, and XPS analysis, enabling comparison of Fe³⁺/Fe²⁺ ratios before reduction, after reduction, and during reoxidation.

**1.3.4 Chemical-driven transient, dissipative stiffness properties of Fe^3+/2+^-CMCMA cryogel****.**

The dissipative, transient mechanical properties of redox-responsive Fe³⁺-CMCMA cryogel were evaluated by rheometry using a temperature-controlled parallel-plate configuration (20 mm diameter titanium plates) at a fixed temperature of 20 °C and fixed gap mode. Measurements were performed at a frequency of 1 Hz and a strain of 1%. The gels were immersed in 2 mL of ascorbate solutions at different concentrations (5 mM, 10 mM, and 20 mM) prepared in MES buffer (pH 7.2) for 5 min and then washed three times using MES buffer under anaerobic conditions for 5 min, followed by exposure of the sample to aerobic conditions. The mechanical properties were monitored at different time intervals by loading the samples onto the rheometer.

**1.3.5 Chemical-driven transient, dissipative tensile properties of Fe^3+/2+^-CMCMA cryogel****.**

For tensile measurements, Fe³⁺-CMCMA cryogels were treated with ascorbate solutions under the same conditions described above, followed by rinsing three times with buffer to remove residual reductant. The samples were then purged with nitrogen to fix the Fe^3^⁺/Fe^2^⁺ redox state and subjected to uniaxial tensile testing at a constant crosshead speed of 1 mm min^-^¹. Tensile tests were performed at different time points after treatment with ascorbate to obtain time-dependent stress-strain curves. The Young’s modulus was determined from the slope of the linear region of the corresponding stress-strain curves.

**1.3.6 Photochemical-driven transient, dissipative stiffness properties of PS-I-loaded Fe^3+/2+^-CMCMA cryogel****.**

The PS-I-loaded Fe³⁺-CMCMA cryogels were immersed in the buffer described above and kept in the dark prior to measurements. The mechanical properties were evaluated using a rheometer with a temperature-controlled parallel-plate configuration (20 mm-diameter titanium plates) at 20 °C and a fixed-gap mode, at a frequency of 1 Hz and a strain of 1%. The gel samples immersed in the PS-I-containing buffer were irradiated using a xenon lamp equipped with a 400 nm band-pass filter at an intensity of 50 mW cm^-^² for different time intervals (20-40 min). Immediately after light irradiation, the gels were kept in the dark, and the mechanical properties were recorded at different time intervals by loading the samples onto the rheometer.

**1.3.7 Photochemical-driven transient, dissipative tensile properties of PS-I-loaded Fe^3+/2+^-CMCMA cryogels****.**

For tensile measurements, PS-I-loaded Fe³⁺-CMCMA cryogels were immersed in the buffer described above and kept in the dark prior to testing. The samples were irradiated using a xenon lamp equipped with a 400 nm band-pass filter (50 mW cm^-^²) for defined time intervals, followed by nitrogen purging to fix the Fe^3^⁺/Fe^2^⁺ redox state. Uniaxial tensile tests were then performed at a constant crosshead speed of 1 mm min^-^¹ to obtain time-dependent stress-strain curves. The Young’s modulus was determined from the slope of the linear region of the corresponding stress-strain curves.

**1.3.8 Calculation of Young’s modulus from tensile stress-strain curves.**

Engineering stress-strain curves were obtained from uniaxial tensile tests^2^. Engineering stress (σ), **equation** **1,** was calculated as

**equation** **1:** $\sigma=\frac{F}{A_{0}}$

where F is the applied force, and the initial cross-sectional area was **A_0_ = 36 mm²**.

Engineering strain (ε), **equation** **2,** was calculated as

**equation** **2:** $\varepsilon=\frac{\Delta L}{L_{0}}$

where ΔL is the extension, and the initial gauge length was **L_0_ = 20 mm**.

The Young’s modulus (E), **equation** **3,** was determined from the slope of the linear elastic region of the corresponding stress-strain curves.

**equation** **3:** $E=\frac{d\sigma}{d\varepsilon}$

**1.3.9 Preparation of insulin-FITC conjugates****.**

Insulin-FITC conjugates were prepared following a standard isothiocyanate labeling procedure by G. sitta Sittampalam et.al.^3^ Briefly, insulin was dissolved in carbonate buffer (pH 8.3) and reacted with fluorescein isothiocyanate (FITC, λ_ex_=490 nm, λ_em_=525 nm) under gentle stirring in the dark for 24 hours. After completion of the reaction, excess unreacted FITC was removed and washed using centrifugal ultrafiltration units (3 kDa) for at least 3 times. The purified insulin-FITC conjugates were collected and stored at 4 °C in the dark prior to use.

**1.3.10 Loading of Fe³⁺-CMCMA cryogels with fluorophore-labeled** **conjugates****.**

Fluorophore-labeled conjugates were loaded into Fe³⁺-CMCMA cryogels during gel formation by introducing the conjugates into the CMCMA precursor solution prior to cryopolymerization. TMR-D (10 μg mL^-^¹), insulin-FITC (10 μM), or Cy3-labeled anti-VEGF aptamer (1 μM) was added to the precursor solution before gel fabrication as described in Section 1.3.1. After gel formation and iron crosslinking, the cryogels were washed and stored in MES buffer to remove non-entrapped conjugates prior to use.

**1.3.11 Chemical-driven transient, dissipative load-release**.

For monitoring chemically driven load release, disc-shaped Fe³⁺-CMCMA cryogels loaded with fluorophore-labeled conjugates were incubated in 1 mL of ascorbate solutions (5, 10, or 20 mM) prepared in MES buffer (pH 7.2) for 5 min. The gels were then washed three times with the same buffer to remove residual ascorbate and transferred into fresh buffer. Aliquots of the supernatant were collected at 10 min intervals, and fluorescence spectra were recorded using the following excitation and emission wavelengths: Cy3-labeled anti-VEGF aptamer (λ_ex_=554 nm, λ_em_=568 nm), insulin-FITC (λ_ex_=490 nm, λ_em_=525 nm), and TMR-D (λ_ex_=555 nm, λ_em_=580 nm). The amount of released conjugates was quantified using a calibration curve obtained under identical buffer conditions.

**1.3.12 Photochemical-driven transient, dissipative load-release**.

For monitoring photochemically driven load release, disc-shaped Fe³⁺-CMCMA cryogels loaded with fluorophore-labeled conjugates were immersed in 1 mL of the buffer described above. The samples were irradiated using a xenon lamp equipped with a 400 nm band-pass filter at an intensity of 50 mW cm^-^² for different time intervals (20-40 min), followed by storage in the dark. Fluorescence spectra of the supernatant were collected every 10 min using appropriate excitation and emission wavelengths for each fluorophore. The amount of released conjugates was quantified using a calibration curve generated from corresponding fluorophore-labeled conjugates prepared in the same buffer.

**1.3.13 Bilayer device fabrication**.

For the synthesis of the bilayer device, a nitrogen-purged water solution (120 μL) comprising N-isopropylacrylamide (NIPAM), N, N’-methylenebisacrylamide (bis-AAm), and ammonium persulfate (APS) was injected into a rod-shaped Teflon mold (40 mm length, 4 mm width, 8 mm height), followed by the addition of 7.4 μL of TEMED under nitrogen (final concentrations: 0.5 M NIPAM, 2.5 mM bis-AAm, 17.5 mM APS, and 35 mM TEMED). The mold was covered with Parafilm and degassed with nitrogen for 7 min to allow APS initiation, sealed, and stored at -18 °C for 2 h to form the pNIPAM layer. After gelation, the gel was thawed at room temperature for 30 min under anaerobic conditions and washed three times with deionized water. Subsequently, a CMCMA solution (2% w/w) containing APS (16.6 mM) was added onto the pNIPAM layer, degassed with nitrogen for 5 min, followed by the addition of TEMED (33 mM) yielding a final total volume of 600 μL. The mold was immediately placed at -20 °C for 4 h to induce cryopolymerization of the CMCMA layer. The resulting bilayer cryopolymer was then immersed in 20 mL of an aqueous Fe²⁺ solution (40 mM) prepared in MES buffer (pH 7.2) and sealed under an anaerobic atmosphere for 24 h to allow sufficient coordination of iron ions with the carboxylate groups of CMCMA. Then, the samples were incubated under ambient conditions for 3 days, allowing spontaneous oxidation and formation of the iron-crosslinked CMCMA layer. The linear bilayer pNIPAM/Fe³⁺-CMCMA device was removed from the mold, washed with MES buffer, and stored in buffer prior to use. The fabrication of the pNIPAM/PS-I-loaded Fe³⁺-CMCMA bilayer device followed the same procedure described above.

**1.3.14 Chemical-driven bending of the bilayer device.**

For chemical-driven bending experiments, the linear pNIPAM/Fe^3+^-CMCMA bilayer device was first transferred from a room-temperature solution into a MES buffer (pH 7.2) solution preheated to 38 °C, where the device bent and reached a stable thermally induced bent configuration. Subsequently, ascorbate (20 mM) was added to the 38 °C solution to chemically trigger further bending of the bilayer device. After 10 min treatment, the device was washed with fresh MES buffer at 38 °C to remove residual ascorbate and was maintained under aerobic conditions until reoxidation returned the device to the thermally induced bent state. Finally, the bilayer device was transferred back into a MES buffer at room temperature, allowing it to fully recover to its original linear configuration.

**1.3.15 Photochemical-driven bending of the bilayer device.**

For photochemical-driven bending experiments, the linear pNIPAM/PS-I-loaded Fe^3+^-CMCMA bilayer device was first transferred from a room temperature solution into MES buffer (pH 7.2) preheated to 38 °C, where the device bent and reached a stable thermally induced bent configuration. Subsequently, the bilayer device was irradiated using a xenon lamp equipped with a 400 nm band-pass filter at an intensity of 50 mW cm^-2^ for 20 min to photochemically trigger further bending. After irradiation, the device was maintained in fresh MES buffer at 38 °C under aerobic conditions until the bilayer returned to the thermally induced bent state. Finally, the bilayer device was transferred back to a MES buffer at room temperature, allowing it to recover to its original linear state.

**2. Rheometry characterization of Fe^3^⁺^/2+^-CMCMA cryogels and hydrogels before and after reduction**

**Figure S1** depicts the low-shear oscillatory rheometry measurements following the time-dependent storage modulus (G’) and loss modulus (G’’) of Fe³⁺-CMCMA cryogel (**Figure S1 (A)**) and Fe^3+/2+^-CMCMA hydrogel (**Figure S1 (B)**). Reduction was performed by incubating the gels in a 20 mM ascorbate solution for 5 min, followed by three washes with buffer, after which the rheological measurements were immediately conducted. Curves labeled S₀ and S₁ correspond to the storage modulus (G’) before and after ascorbate reduction, respectively, while curves labeled S₀’ and S₁’ correspond to the loss modulus (G’’) before and after reduction, respectively. In addition, cyclic reduction/oxidation is presented in Figure S1 (C).


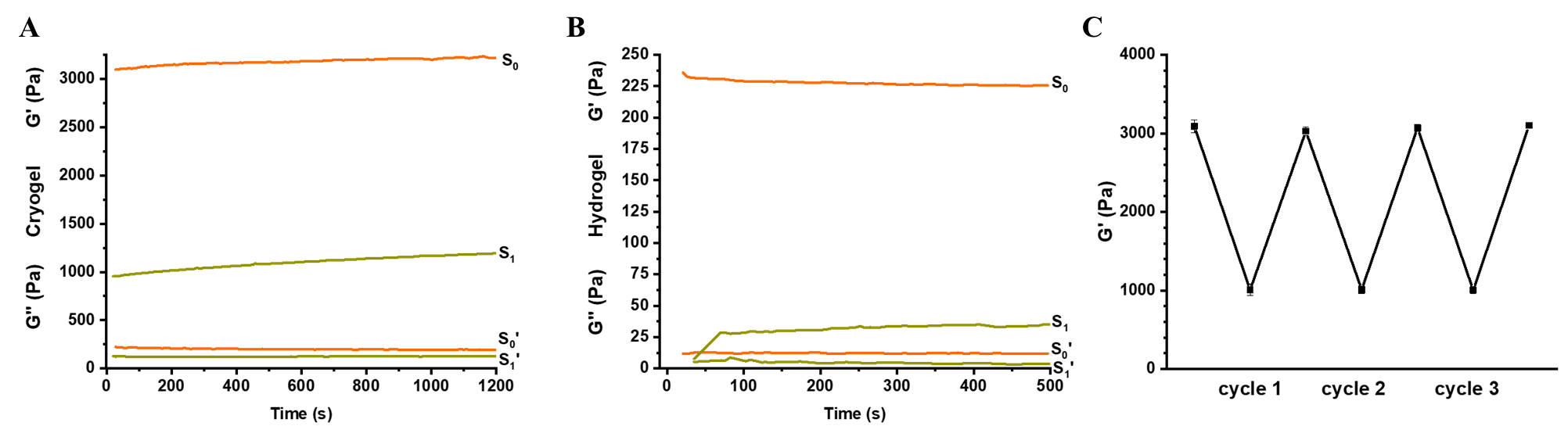


**Figure S1**. Storage modulus (G') and loss modulus (G'') corresponding to: **(A)** Fe³⁺-CMCMA cryogel, and **(B)** hydrogel, before (S_0_) and after (S_1_) ascorbate-induced (20 mM) reduction measured under oscillatory shear. **(C)** *G*′ changes of the Fe³⁺/Fe^2^⁺-CMCMA upon the cyclic reduction/oxidation of the cryogel.

Table S1. G’ values of the corresponding cryogel and hydrogel before and after ascorbate-induced reduction.

| **State** | **Cryogel** | | **Hydrogel** | |
| --- | --- | --- | --- | --- |
|  | **G’** | **G’’** | **G’** | **G’’** |
| **Fe^3+^-CMCMA (S_0_)** | **3092** | **221** | **225** | **12** |
| **Fe^2+^-CMCMA (S_1_)** | **1007** | **123** | **25** | **3** |

The temporal variations of the storage modulus (G’) of the Fe^3+^-CMCMA hydrogel subjected to ascorbate treatment at different concentrations are shown in **Figure S2 (A)**. The data provide a comparison of the cryogel framework with the time-dependent mechanical response of the hydrogel systems during the redox process, **Figure S2 (B)**.


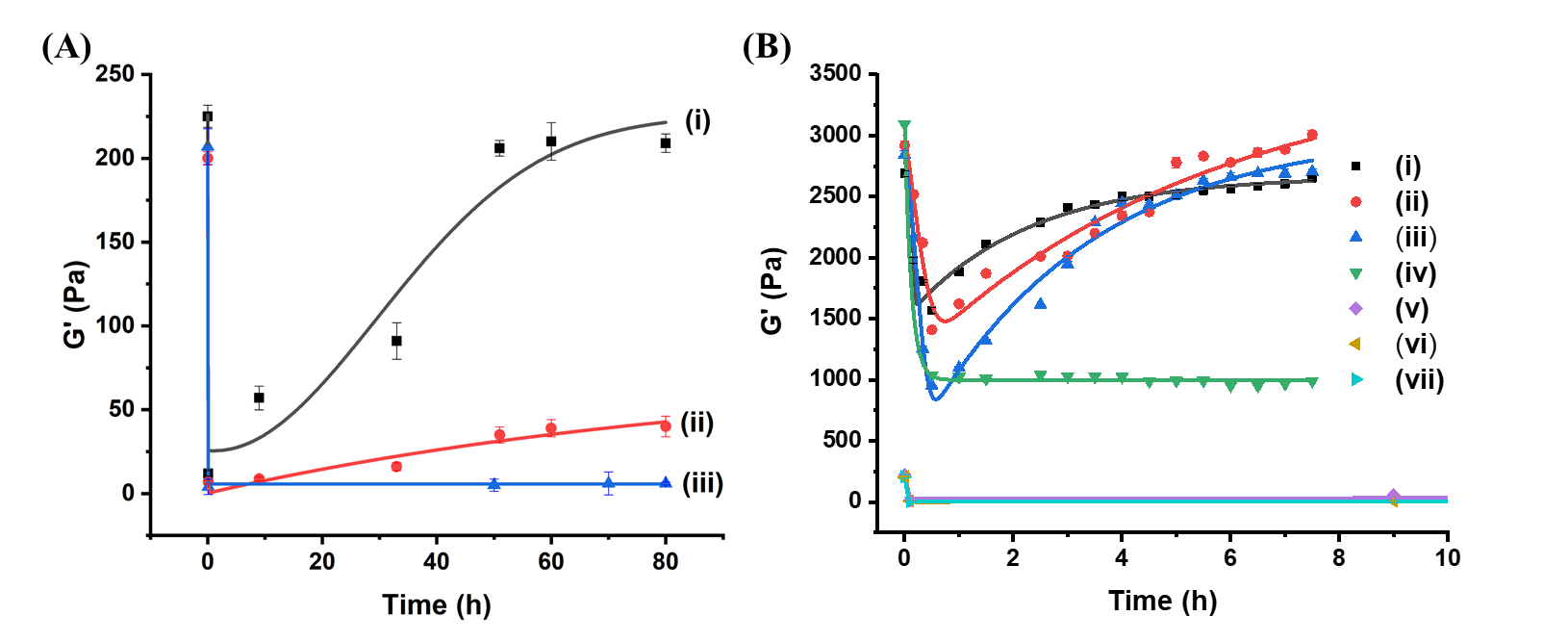


**Figure S2**. **(A)** Time-dependent storage modulus (G’) of Fe³⁺-CMCMA hydrogels treated with ascorbate at concentrations corresponding to: **(i)** 5 mM, **(ii)** 10 mM, and **(iii)** 20 mM, under aerobic conditions. **(B)** Time-dependent storage modulus (G’) comparison of Fe³⁺-CMCMA hydrogels and cryogels treated with ascorbate at concentrations corresponding to: Cryogels - **(i)** 5 mM, **(ii)** 10 mM, **(iii)** 20 mM under aerobic conditions and **(iv)** 20 mM under N_2_ condition, and hydrogels - **(v)** 5 mM, **(vi)** 10 mM, **(vii)** 20 mM, under aerobic conditions.

**3. XPS analysis: quantification of Fe³⁺/Fe²⁺ ratio in transient, dissipative Fe³⁺-CMCMA cryogel, for a detailed analysis of the Fe³⁺/Fe²⁺ ratio and the deconvolution process implemented to quantify the transient Fe³⁺/Fe²⁺ ratio.**


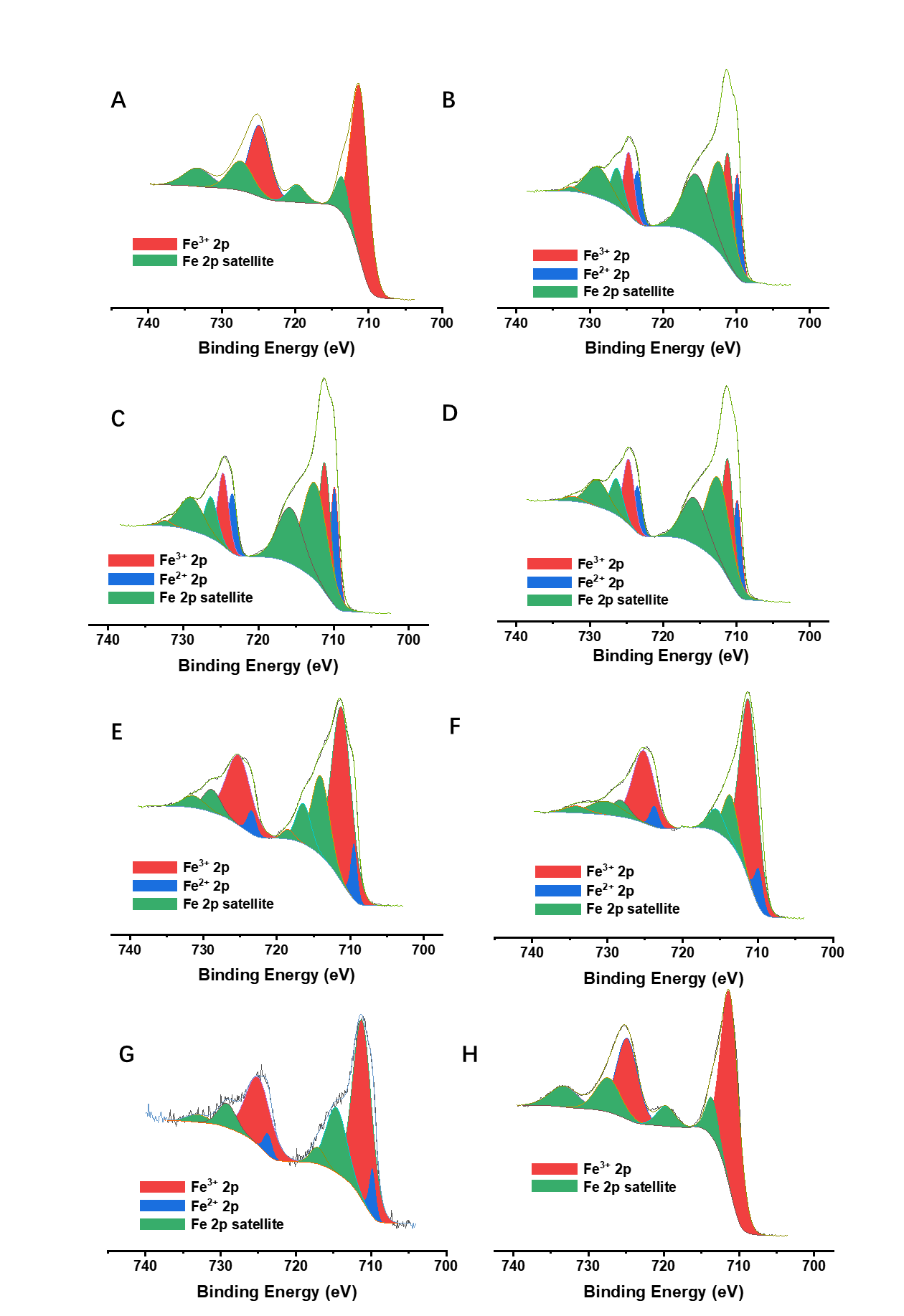


**Figure S3.** High-resolution Fe 2p XPS spectra of Fe³⁺-CMCMA gels recorded before reduction **(A)** and after reduction followed by reoxidation for 0 h **(B)**, 1 h **(C)**, 2 h **(D)**, 6 h **(E)**, 8 h **(F)**, 10 h **(G)**, and 12 h **(H)** under aerobic conditions.

X-ray Photoelectron spectroscopy (XPS) measurements were performed using a Kratos AXIS Supra spectrometer (Kratos Analytical Ltd., Manchester, U.K.) with Al Kα monochromatic radiation X-ray source (1486.6 eV) X-ray beam from the Al target was 15 kV, 15 mA. The XPS spectra were acquired with a takeoff angle of 90° (normal to the analyzer); vacuum condition in the chamber was 2×10^-9^ torr. High-resolution XPS spectra of Fe 2p were measured with a pass energy of 20 eV and 0.1 eV step size. The binding energies were calibrated using the C 1s peak energy of 285.0 eV. Data was collected and analyzed by using ESCApe processing program (Kratos Analytical Ltd.) and Casa XPS (Casa Software Ltd.). The transient redox behavior of the Fe³⁺-CMCMA cryogel was investigated by time-dependent XPS. High-resolution Fe 2p spectra were recorded at different time points following chemical reduction and subsequent exposure to aerobic conditions shown in **Figure S3(A-H)**. XPS interpretation of Fe^3+^ and Fe^2+^ species and quantitative evaluation of the species based on analyzing the Fe 2p core-level binding energies, spin-orbit splitting, and satellite peak characteristics^1-3^. The Fe 2p spectrum splits into two primary spin-orbit coupling components: Fe2p3/2 and Fe2p1/2 (Δoxide = 13.6 eV). Fe 2p region has significantly split spin-orbit components with an intensity ratio of about 2/1. Each part consists of a main peak and a “shake-up” satellite. Shake-up satellites arise when a photoelectron transfers energy to a valence electron, exciting it to a higher unoccupied level. This appearance is highly indicative of specific oxidation states. The main Fe^2+^ 2p3/2 peak typically appears at a binding energy level of roughly 709.5 eV to 710.0 eV. The main Fe3+ 2p3/2 peak is found at a slightly higher binding energy of about 710.5 to 711.5 eV. Satellite structures are crucial for confirming oxidation states because they arise from different final-state screening effects after an electron is ejected. Fe^2+^ Satellite features a strong, distinct satellite peak located at a binding energy of about 715.5 eV to 716 eV. Fe^3+^ features a broader, less intense satellite peak located at a higher binding energy around 718 eV to 719 eV.  The Shirley background was applied across the entire Fe 2p region 700 - 736 eV. The background endpoint includes the higher-energy shake-up satellite regions, as their area contributes to the overall ion concentration, and the Gaussian-Lorentzian GL (30) ratio was applied to determine peak shape. Iron oxides are typically insulating or semiconducting. The flood gun charge-neutralizing and calibration, and the final binding energy scale to the adventitious carbon C 1s at 285.0 eV, a reference for charge correction, was used. The time-dependent variation in the Fe³⁺/Fe²⁺ ratio reflects the transient redox state of the iron crosslinkers within the cryogel network.

1. Yamashita, T.; Hayes, P. Analysis of XPS Spectra of Fe²⁺ and Fe³⁺ Ions in Oxide Materials. *Appl. Surf. Sci.* **2008**, 254, 2441–2449.

2. Baretta, R.; Davidson-Rozenfeld, G.; Gutkin, V.; Frasconi, M.; Willner, I. Chemical and Photochemical-Driven Dissipative Fe³⁺/Fe²⁺-Ion Cross-Linked Carboxymethyl Cellulose Gels Operating Under Aerobic Conditions: Applications for Transient Controlled Release and Mechanical Actuation. *J. Am. Chem. Soc.* **2024**, 146, 9957–9966.

3. Derriere, R.; Maccario, M.; Croguennec, L.; Le Cras, F.; Delmas, C.; Gonbeau, D. X-Ray Photoelectron Spectroscopy Investigations of Carbon-Coated LiₓFePO₄ Materials. *Chem. Mater.* **2008**, 20, 7164–7170.

**4. Calibration curves for fluorophore-labeled conjugates**

The amounts of released fluorophore-labeled conjugates were quantified using calibration curves obtained by correlating fluorescence intensities with the concentrations of the corresponding conjugates under identical buffer conditions to those used in the release experiments.


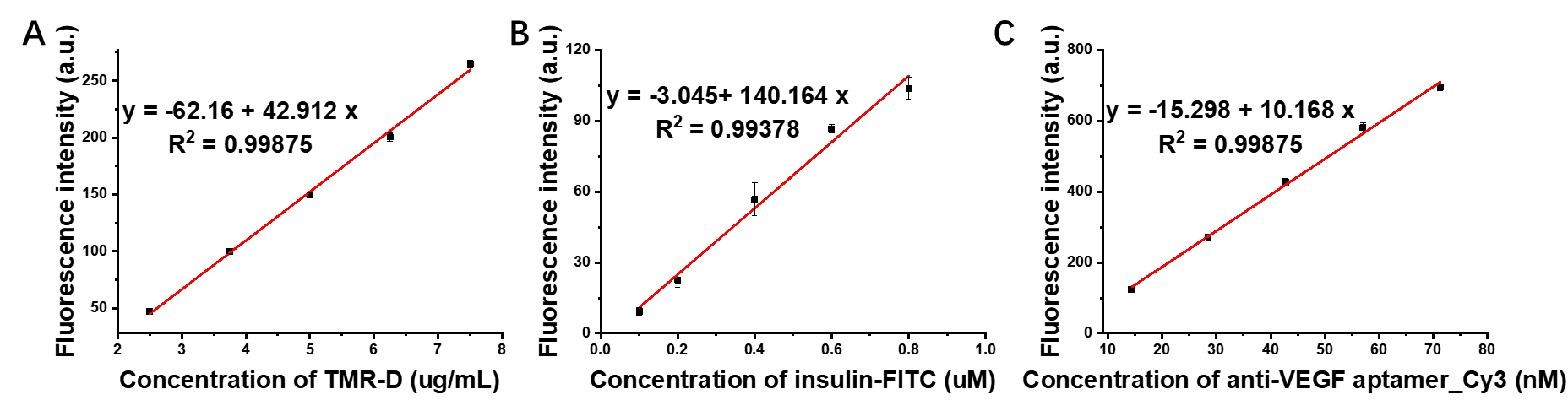


**Figure S4.** Calibration curves of **(A)** TMR-D, **(B)** insulin-FITC, and **(C)** Cy3-labeled anti-VEGF aptamer.

**5. Quantification of the fluorophore-labeled conjugates ratio**

Following the reported method^4^, a calibration curve of FITC was established from its UV-vis spectra, and the absorbance values at 495 nm and 280 nm were extracted for each concentration point as shown in **Figure S5**.


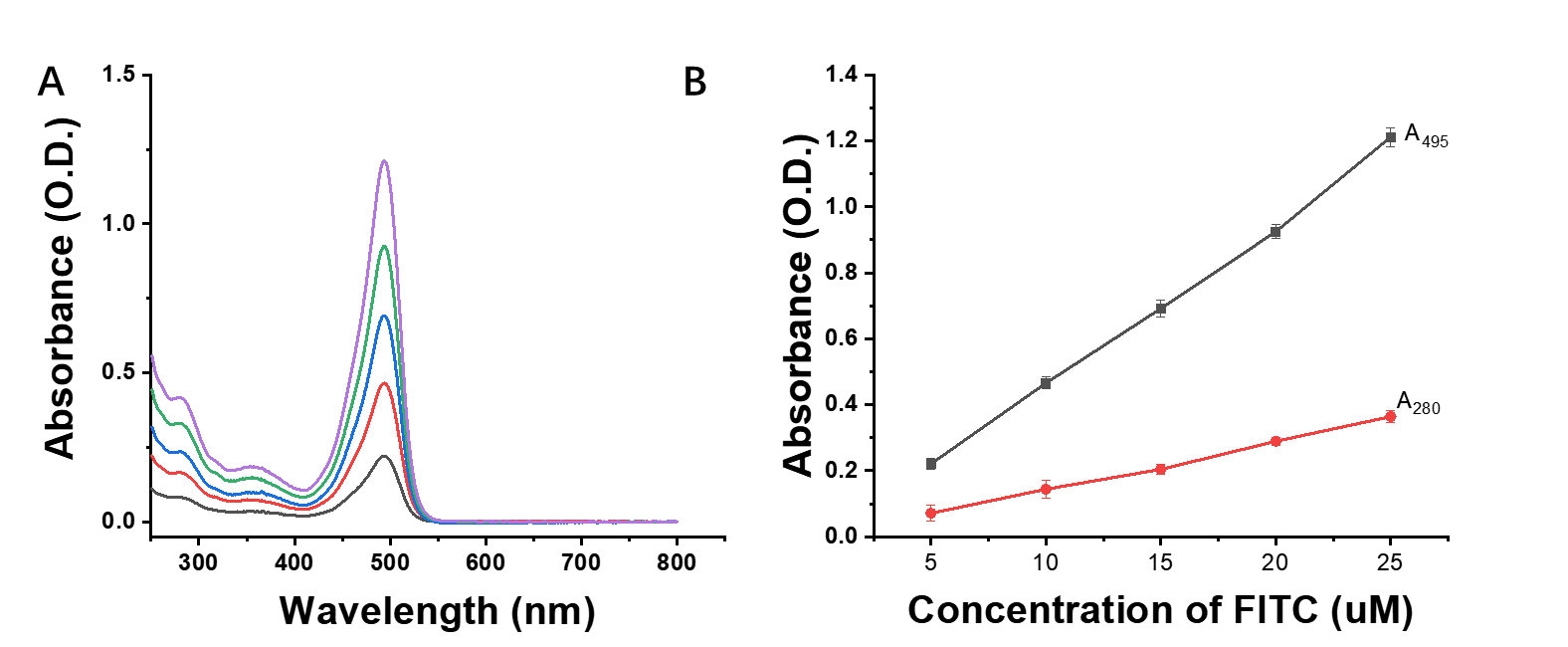


**Figure S5**. UV-vis absorption spectra of FITC at different concentrations **(A)** and corresponding calibration plots of absorbance at 495 nm and 280 nm as a function of FITC concentration **(B)**.

The ratios of A₄₉₅/A₂₈₀ obtained at different concentrations were averaged, yielding a correction factor of 3.25426 (**Table S2**).

**Table S2.** Absorbance values of FITC at 495 nm (A₄₉₅) and 280 nm (A₂₈₀) at different concentrations, and the corresponding A₄₉₅/A₂₈₀ ratios used to calculate the average correction factor for FITC.

| Concentration of FITC (μM) | A_495_ | A_280_ | A_495_/A_280_ |
| --- | --- | --- | --- |
| 5 | 0.221 | 0.071 | 3.11268 |
| 10 | 0.466 | 0.144 | 3.23611 |
| 15 | 0.692 | 0.204 | 3.39216 |
| 20 | 0.925 | 0.289 | 3.20069 |
| 25 | 1.212 | 0.364 | 3.32967 |
| Average of A_495_/A_280_ | | | 3.25426 |

Based on the measured absorbance A₄₉₅ = 0.584, the contribution of FITC to the absorbance at 280 nm was estimated to be 0.584/3.25426 = 0.17946. Subtracting this value from the measured A₂₈₀ = 0.194 gave an insulin-related absorbance of 0.01454 O.D. The FITC-to-insulin labeling ratio was therefore calculated as 0.17946 / 0.01454 ≈ 12.34, indicating that, on average, approximately 12.34 FITC molecules were conjugated to each insulin molecule.


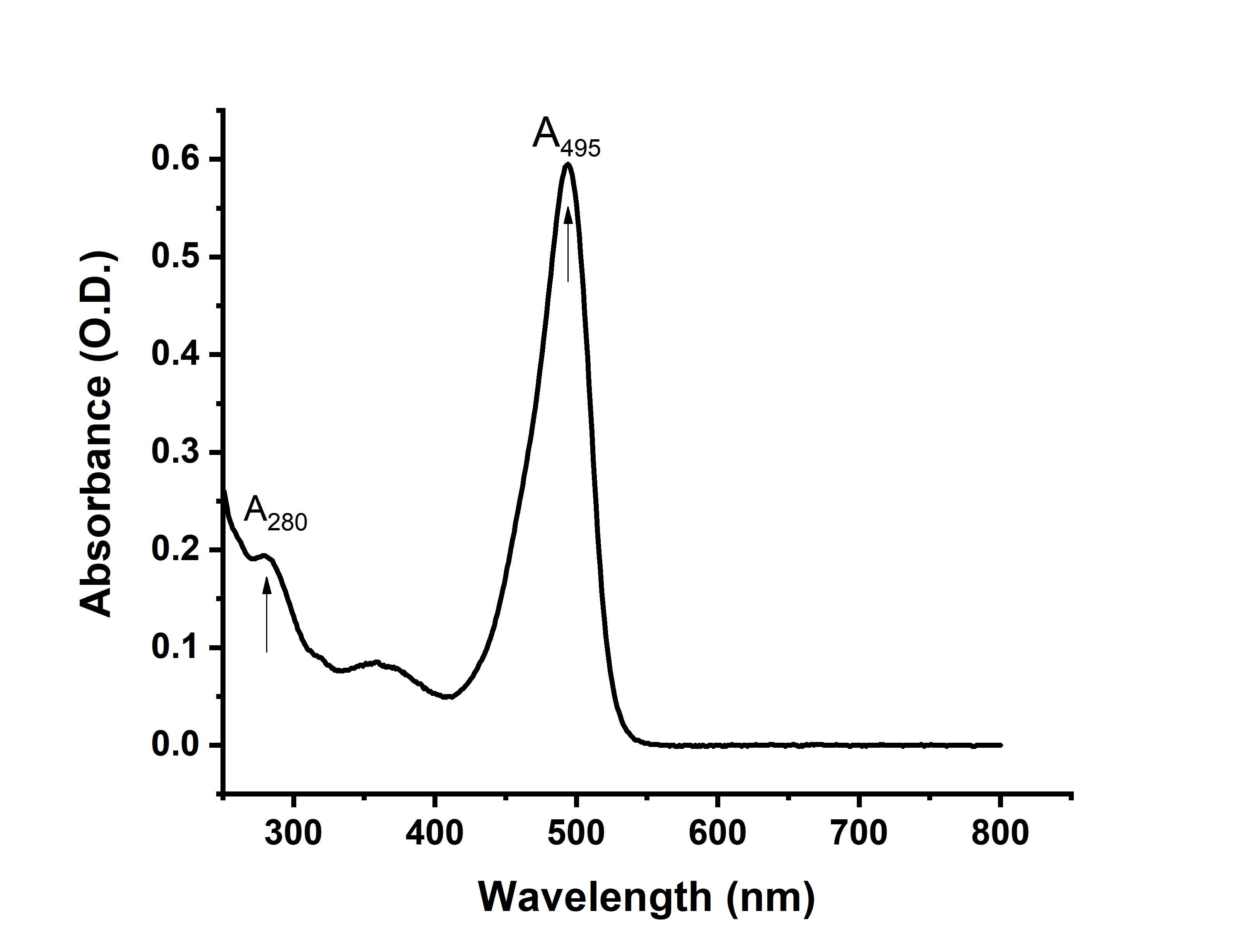


**Figure S6.** UV-vis absorption spectrum of insulin-FITC.

**6. UV-Vis absorption spectra of PS-I**


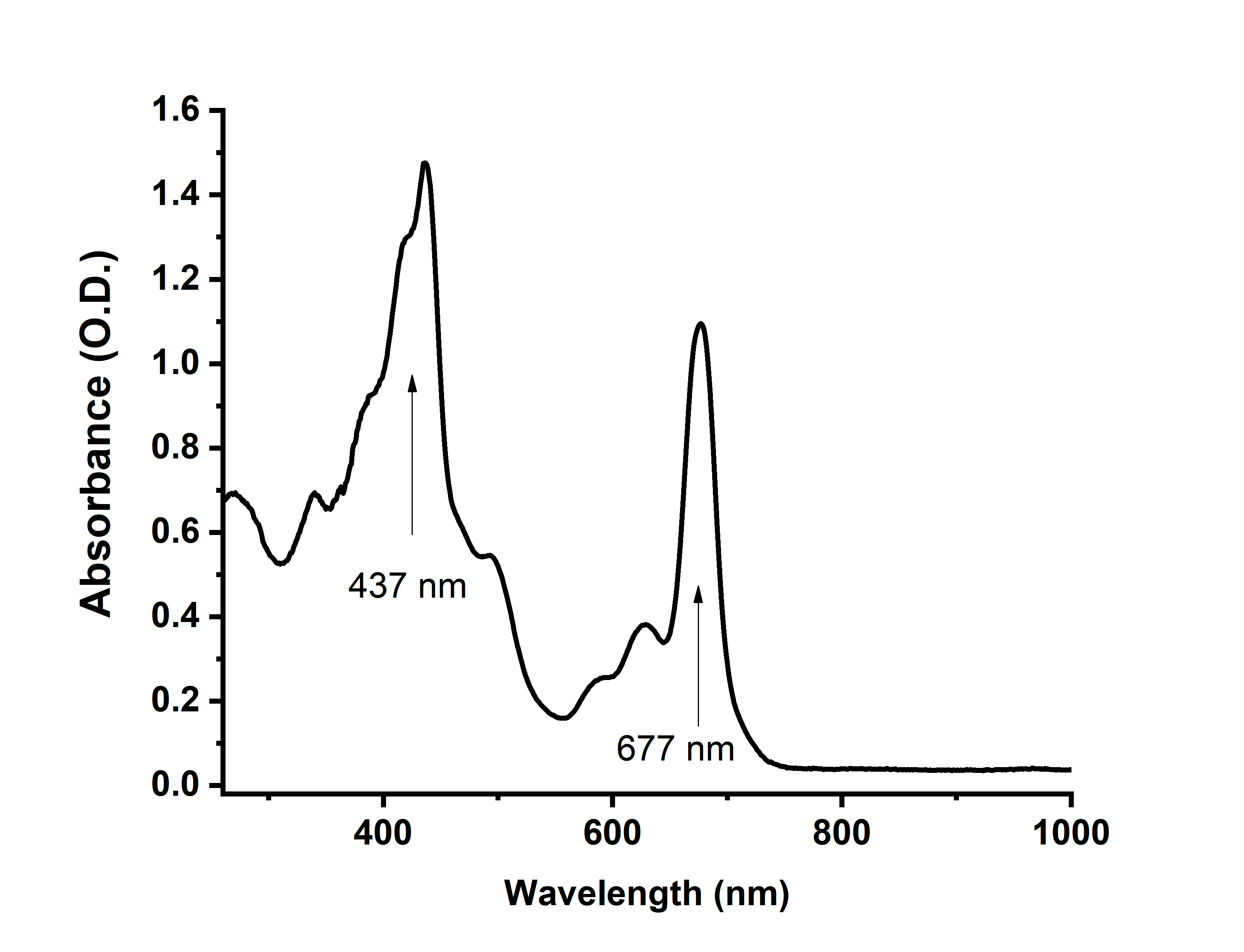


**Figure S7.** UV-vis absorption spectrum of PS-I at a concentration of 0.25 mg mL^-^¹, revealing characteristic absorption maxima at 437 nm and 677 nm.

**7. Bending of bilayer devices and the transient curvature characterization**

The temporal bending curvatures of the bilayer devices were calculated using **Equation** **4**^5^ as stated in the main text. After 2 min incubation in 38 °C, the bilayer device undergoes a transition from a linear configuration to a bent configuration, exhibiting a curvature (*1/r*) of approximately 0.09 mm^-^¹. When maintained at 38 °C, the bilayer device maintains its bent configuration for up to 10 min, with only minor variations in curvature. Upon cooling the solution to 25 °C, the bent bilayer device gradually relaxes to a less curved configuration and progressively restores its parent linear state within approximately 160 min (**Figure S8 (A)**). The corresponding time-dependent curvatures of the bilayer device during the heating and cooling processes are summarized in **Figure S8 (B)**.

**equation** **4:** $r=\frac{y^{2}}{8x}+\frac{x}{2}$


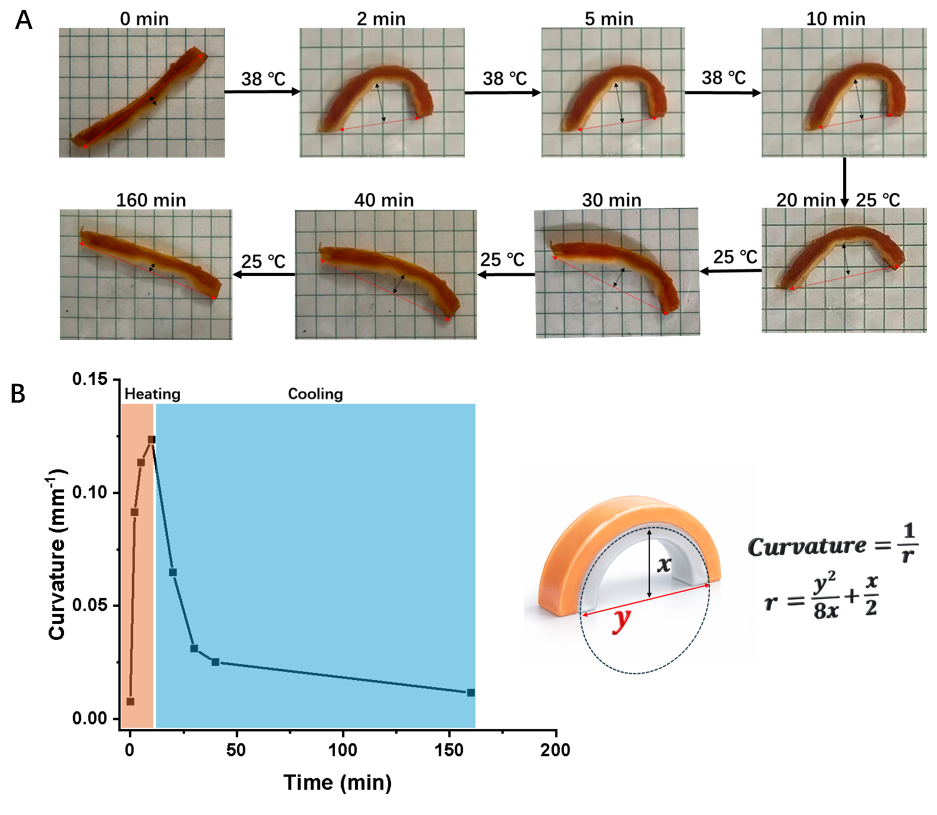


**Figure S8. (A)** Images corresponding to the time-dependent mechanical bending of the pNIPAM/Fe³⁺-CMCMA bilayer device upon thermal triggering by heating the device to 38 °C, followed by cooling to 25 °C and recovery to the linear configuration. **(B)** Corresponding curvature of the pNIPAM/Fe³⁺-CMCMA bilayer device as a function of time during the heating and cooling processes and the schematic evaluation of the bending degree.

**8. Reference**

[1] Caspy, I., Neumann, E., Fadeeva, M. *et al.* Cryo-EM photosystem I structure reveals adaptation mechanisms to extreme high light in *Chlorella ohadii*. *Nat. Plants* 7, 1314-1322 (**2021**).

[2] Sun, JY. *et al*. Highly stretchable and tough hydrogels. *Nature* **2012**, 489, 133-136.

[3] Hentz, N. G. Synthesis and Characterization of Insulin-Fluorescein Derivatives for Bioanalytical Applications. *Anal. Chem.* **1997**, *69* (24), 4994-5000.

[4] Sheba Johnson, Wei Liu, *et al.* Surface Chemistry and Spectroscopy of Human Insulin Langmuir Monolayer. *J. Phys. Chem. B* **2012,** 116, 34, 10205-10212.

[5] H. T. H. Piaggio, *Nature*, **1952**, 169, 560
